# Effects of different irrigation lower limits and combined application of reduced nitrogen with organic fertilizer on spring-season tomatoes (*Solanum lycopersicum*)

**DOI:** 10.1371/journal.pone.0354927

**Published:** 2026-08-11

**Authors:** Hai-jie Li, Yuan Huang, Jing-xin Yu, Guo-ying Tian, Xin-na Gao, Yi-fan Kang, Ya-ru Du, Yang-yang Wu, Ying-ru Yang

**Affiliations:** 1 Department of Shijiazhuang Academy of Agricultural and Forestry Sciences, Shijiazhuang, Hebei Province, China; 2 Department of Shijiazhuang Agricultural Information Engineering Technology Research Center, Shijiazhuang, Hebei Province, China; 3 Department of Beijing Academy of Agriculture and Forestry Sciences, Beijing, China; Dev Bhoomi Uttarakhand University, INDIA

## Abstract

Greenhouse tomatoes (*Solanum lycopersicum*) are a high-value cash crop globally; however, inefficient water and nitrogen management in greenhouse systems often leads to resource waste, environmental risks, and reduced yield/quality, thereby posing challenges for sustainable cultivation. Herein, we aimed to optimize water and fertilizer management for spring greenhouse tomatoes by investigating how different irrigation lower limits, combined with reduced nitrogen and organic fertilizer, affect photosynthetic performance, yield, fruit quality, and water**–**nitrogen use efficiency. A three-factor experiment was conducted in 2024. Treatments included two irrigation levels **(L)** [75–90% field capacity (FC**;** L1) and 85–90% FC (L2), where FC refers to the soil field capacity]; two basal organic fertilizer application rates **(O)** [high (O1: 400 kg decomposed sheep manure) and low (O2: 280 kg decomposed sheep manure)]; and three topdressing nitrate**-**nitrogen levels **(N)** [conventional (N1: 352.14 kg·hm ⁻ ²), 20% reduced (N2: 281.72 kg·hm ⁻ ²), and 40% reduced (N3: 211.29 kg·hm ⁻ ²)]. Data were analyzed using three-way ANOVA, principal component analysis (for photosynthetic and quality traits), and entropy-weighted TOPSIS (comprehensive evaluation)**.** The first-level indicators in the system included photosynthetic indicators (F1), yield indicators (F2), quality indicators (F3), water use indicators (F4), partial factor productivity of nitrogen (F5), and nitrogen–phosphorus–potassium accumulation (F6). The results revealed that the treatments L2, N3 and O2 can increase the average yield of tomatoes, and the combination of L2 (85%–90% FC), O2 (low organic fertilizer), and N2 (20% reduced nitrogen) not only displayed the highest levels of vitamin C (21.46 mg/100g) and soluble solids content (5.62%) in fruits but also exhibited the highest total nitrogen content in fruits, stems, and leaves across all treatments**,** surpassing the lowest-performing treatment by 62.83%. Consequently, this treatment yielded the highest comprehensive performance.

## Introduction

Tomato (*Solanum lycopersicum* L.) is one of the most widely cultivated and economically vital vegetable crops globally [1]. China dominates global fresh-market tomato production and ranks among the top three countries in processed tomato output, with Shandong, Hebei, and Henan as its major tomato-cultivating provinces [2,3]. Driven by its nutritional value and versatility, protected cultivation (particularly in greenhouses) has expanded rapidly, enabling year-round production, higher yields, and improved quality by mitigating adverse environments [4,5]. However, intensive greenhouse production often involves excessive irrigation and fertilization, leading to water/nutrient waste, soil degradation, including acidification, and nitrate leaching [6–8], thereby threatening sustainable agriculture.

Optimal irrigation and balanced fertilization are key to addressing these issues, yet critical gaps remain [9,10]. Sensor-based irrigation (e.g., soil moisture control) has improved water use efficiency (WUE): alternate partial root-zone drip irrigation at 70% field capacity (FC) optimized yield/quality in Huaibei [11], while Wang et al. (2024) reported an intelligent fertigation system (IFS, integrating water and nutrient delivery) controlling soil moisture at 80–85% FC was optimal for Shouguang [12]. However, the applicability of such strategies to regions with distinct conditions, e.g., Shijiazhuang and Hebei, remains underexplored. For nitrogen management, combining organic fertilizers, such as composted manure, with reduced chemical nitrogen content sustains yield and soil health [13–15]; however, optimal organic–inorganic ratios and their interactions with irrigation remain unclear. Additionally, Fan et al. and Zhou et al. reported that under fixed water conditions, nitrogen optimization beyond a threshold fails to boost yield—highlighting the need for integrated evaluation via principal component analysis (PCA) and entropy-weighted TOPSIS [16–22].

In this study, we aimed to fill these gaps by investigating the integrated effects of irrigation thresholds, organic fertilization, and nitrogen reduction on greenhouse tomatoes in Shijiazhuang. Specific objectives were to (1) evaluate interactions between irrigation lower limits and basal organic fertilizer levels on tomato photosynthetic traits, yield, and quality; (2) assess the coupling effects of these factors with topdressing nitrogen reduction on water and nitrogen use efficiency; and (3) identify the optimal water**–**fertilizer combination for maximum comprehensive benefits using multi-criteria decision analyses, such as PCA and entropy-weighted TOPSIS.

## Materials and methods

### Experimental site and greenhouse details

This study was conducted at the Shijiazhuang Academy of Agriculture Sciences Experimental Base (114°36′E, 37°37′N). Formal work permission for on-site access was not required for this experiment, as the test site is a public research platform managed by the Shijiazhuang Academy of Agriculture and Forestry Sciences, and the research activities comply with the platform’s default research access regulations. The solar greenhouse used in the experiment had a steel frame structure, measuring 90 m in length, 8.0 m in width, and 4.5 m in height. The thickness of the rear wall was 0.5 m, and the structure was covered with polyethylene film (light transmittance approximately 85%). The greenhouse was equipped with top and bottom ventilation openings, along with two circulation fans installed on the east wall to regulate internal temperature and humidity according to external conditions. The total planting area was 489 m². The planting area was 489 m². The soil at the test site was loam, and the physical properties and nutrient contents of the soil at a depth of 0–40 cm are shown in Table 1.

**Table 1:** Soil physical and chemical properties.

Depth (cm)	Organic matter (g/kg)	Available nitrogen (mg/kg)	Available phosphorus (mg/kg)	Available potassium (mg/kg)	pH	Electrical conductivity (µS/cm)	Soil properties	Sand content/%	Silt content/%	Clay content/%
0–40	5.79	31.65	17.45	326.5	7.24	84.05	Loam	41.30	41.6	18.9

### Test materials and experimental design

**Fig 1:**
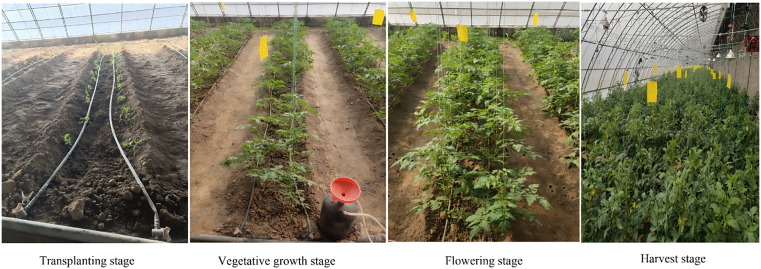
Tomato growth stages. Note: Reprinted from [ref] under a CC BY license, with permission from [Haijie Li], original copyright [2024].

The tomato variety used in the test was ‘Onuo,’ a new pink-fruited tomato cultivar. The plants exhibited vigorous growth, adequate heat tolerance, strong disease resistance, and suitability for cultivation in early spring and summer. The test tomatoes were planted on March 14, 2024, the experiment began on April 1, and the vines were pulled on June 24; thus, the total growth period was 102 days. Fig 1 shows the different stages of tomato production.

The application rates of nitrogen, phosphorus and potassium were determined in line with China’s agricultural industry standard —《Guidance for the Fertilization Control Techniques of Protected Vegetables》(NY/T 3832−2021).The tested organic fertilizer was decomposed sheep manure (N + P₂O₅ + K₂O content ≥4.87%, organic matter content ≥18.4%), and the chemical fertilizers were urea (nitrogen content 46%) and potassium sulfate (K₂O content 52%). According to the experimental design, the total amount of organic fertilizer for different treatments was applied as base fertilizer, and the nitrogen fertilizer was applied five times in equal amounts with irrigation water at different growth stages of tomato, including the reviving stage, flowering and fruit-setting stage, fruit expansion stage, peak fruiting stage, and late harvesting stage. Each plot was applied with an equal amount of potassium fertilizer as the base fertilizer (13.48 kg/hm²). The agricultural management measures between treatments were the same.

In this study, we employed two different irrigation levels (L): 75–90% FC (L1) and 85–90% FC (L2); two base fertilizer levels with high organic matter (O1, 400 kg decomposed sheep manure) and low organic matter (O2, 280 kg decomposed sheep manure); and three topdressing levels using urea (N): conventional nitrogen levels (N1, 352.14 kg/hm²), 20% reduction in nitrogen levels (N2, 281.72 kg/hm²), and 40% reduction in nitrogen levels (N3, 211.29 kg/hm²). The experiment was conducted in a three-factor factorial design, with a total of 12 treatments (Table 2), and each treatment was replicated thrice. Two buffer rows were left between treatments. The cultivation used a wide-narrow row system with planting beds dug 0.2 m deep, 0.6 m wide, and 6.7 m long. The plant spacing was 0.33 m, with narrow rows spaced 0.4 m apart and wide rows 1.1 m apart. Drip irrigation was employed with emitters spaced 0.3 m apart, and one drip tape was laid per row.

**Table 2:** Experimental design, including a total of 12 different treatment groups.

Treatment	Organic fertilizer (O, kg)	Irrigation lower limit (L, %FC)	Nitrogen fertilizer (N, kg/hm²)
**T1**	O1:400	L1:75	N1:352.14
**T2**	O1:400	L1:75	N2:281.72
**T3**	O1:400	L1:75	N3:211.29
**T4**	O1:400	L2:85	N1:352.14
**T5**	O1:400	L2:85	N2:281.72
**T6**	O1:400	L2:85	N3:211.29
**T7**	O2:280	L1:75	N1:352.14
**T8**	O2:280	L1:75	N2:281.72
**T9**	O2:280	L1:75	N3:211.29
**T10**	O2:280	L2:85	N1:352.14
**T11**	O2:280	L2:85	N2:281.72
**T12**	O2:280	L2:85	N3:211.29

Note(s): FC, Field capacity. Treatment codes: T1 = O1L1N1, T2 = O1L1N2,..., and T12 = O2L2N3.

Soil moisture content (volumetric fraction) was monitored using a multi-profile 3D soil moisture monitoring device (Agricore Technology, China). The device was installed at the midpoint of each plot’s north-south axis, 15 cm away from tomato plants on the ridges. According to the technical specifications of NY∕T 3678–2020 (Determination of Field Capacity of Soil by Enclosure Flooding Instrument Method), the maximum water-holding capacity (volumetric fraction) of the tested soil was calculated. The field capacity (hereinafter referred to as FC) of the 0–40 cm soil layer in each plot was measured,Among them, the field capacity of treatments T1–T3 was 41.34%, that of T4–T6 was 38.74%, T7–T9 was 38.27%, and T10–T12 was 39.62%.The monitoring data were transmitted in real time to the management platform (http://210.12.220.75:15002/login). Specifically, the irrigation control module integrated in the platform establishes a stable network connection with the irrigation controller. The irrigation activation condition, i.e., the lower irrigation threshold, is configured through the platform; when the data monitored by the sensors reach the preset parameters, the platform sends an irrigation command to the irrigation controller, and the irrigation process will be terminated automatically once the upper irrigation threshold (90% FC) is achieved.

### Measurement items

#### Environmental conditions and water consumption in solar greenhouse.

**Fig 2:**
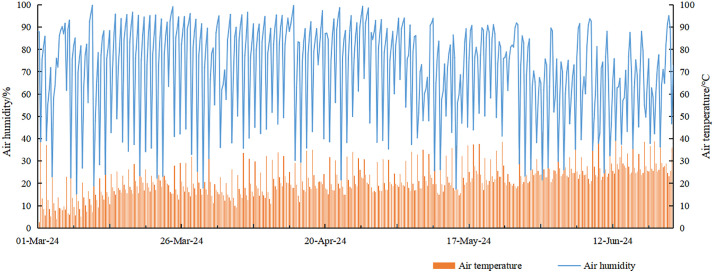
Air temperature and humidity in the solar greenhouse used in this study.

**Fig 3:**
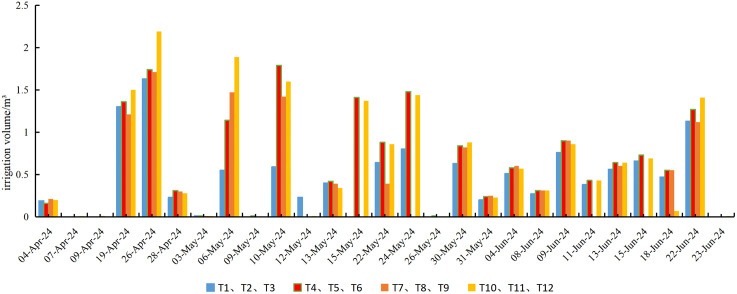
Irrigation volume statistics.

The Greenhouse Doll II (Agricore Technology, China) was used to record air temperature and humidity, with measurements taken once every hour during the experiment (Fig 2). Water meters were installed in each plot to record the total irrigation volume (m³) throughout the growth period, and the water consumption per tomato plant (m³/plant) was calculated based on the planting density (Fig 3).

#### Measurement of photosynthetic parameters.

During the peak fruiting stage (75 days after planting, May 28, 2024, a sunny day), from 9:00–12:00 h, a Li-6800 photosynthesis measurement system (LI-COR, USA) equipped with a fluorescence leaf chamber was used to measure parameters such as net photosynthetic rate, stomatal conductance, and transpiration rate. The third fully expanded functional leaf from the growth point was selected for measurement.

#### Yield and quality indicators.

During the peak fruiting stage of tomatoes (June 4, 2024; June 8, 2024; June 18, 2024), fruits were harvested three times and weighed immediately to record the weight of each plot. Five tomato fruits from the middle node of plants in each plot were collected during the peak fruiting stage for quality index determination. Soluble sugar content (mg·g^−1^) was measured by anthrone colorimetry; titratable acid content (%) was determined by acid-base titration; vitamin C (VC) content (mg·100 g^−^1) was measured by potassium iodate titration; and soluble solids (%) were detected by digital refractometer.

#### Plant nitrogen, phosphorus, and potassium contents.

At the harvest stage, three plants with consistent growth were selected from each treatment and were excavated, keeping their aboveground parts intact. After blanching at 105°C, the roots, stems, and leaves were dried at 80°C, and the fruits were dried at 50°C, until constant weight was achieved. Biomass (kg) was weighed using a balance with 0.01 g weighing accuracy. After grinding and mixing each part, plant nitrogen content (%) was determined by the Kjeldahl method, phosphorus content (%) by spectrophotometry, and potassium content (%) by flame photometry. The accumulation of each component was calculated accordingly.

#### Water and nitrogen use efficiency.

The recorded yields were converted to per-plant yields, and the irrigation volumes measured by water meters were converted to per-plant irrigation volumes. WUE was calculated based on yield and irrigation volume using Equation (1) as follows:


$$\begin{array}{c} WUE=Y/I_{total}\text{,} \end{array}$$ (1)

where *Y* is the yield per plant (kg/ plant), and *I*_*total*_ is the water consumption per plant (m³/ plant).

The partial factor productivity of nitrogen fertilizer (NPP) was calculated based on yield and topdressed nitrogen using Equation (2) as follows:


$$\begin{array}{c} NPP=\;Y/N, \end{array}$$ (2)

where *N* is the nitrogen application rate (kg/ plant).

### Data analyses

**Fig 4:**
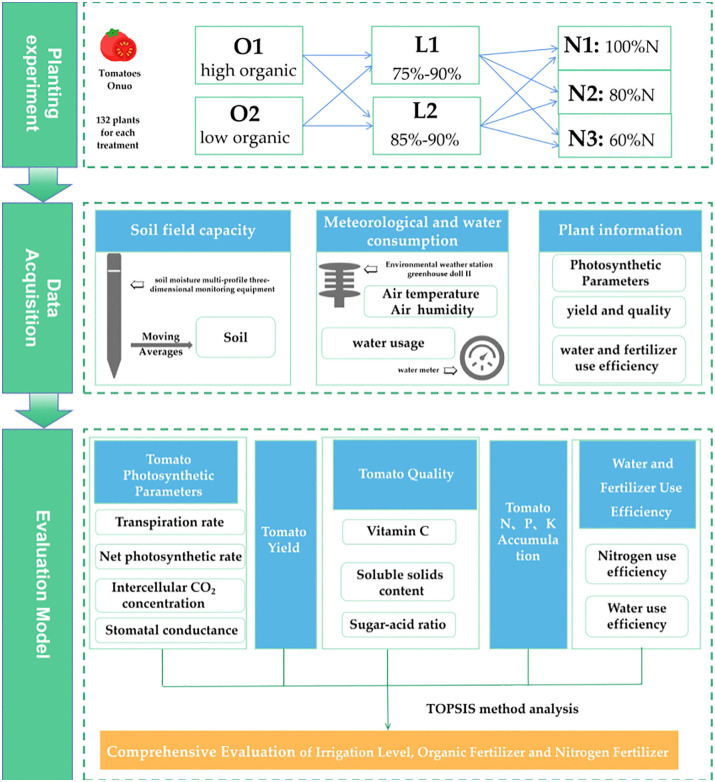
Flowchart depicting the experimental design.

Excel was used for data tabulation. IBM SPSS 23 software was employed for multivariate statistical analysis. A three-way analysis of variance was performed to determine significant differences between data sets. Spearman’s correlation coefficients (*P* < 0.05) were calculated for all indicators. All indicators were normalized, and PCA was used to evaluate tomato traits. The entropy-weighted TOPSIS method was applied for comprehensive ranking. The experimental design is presented in Fig 4.

PCA was employed to reduce the dimensionality of photosynthetic characteristics and fruit quality indicators and to obtain integrated scores. The calculation procedure used is described below.

#### Data standardization.

The original data matrix $\text{X}={\left({\text{x}}_{\text{ij}}\right)}_{n\times m}$ was standardized to eliminate dimensional effects using Equation (3) as follows:


$$\begin{array}{c} {\text{x}}_{ij}^{\text{'}}=\frac{x_{ij}-x_{j}}{n_{j}}\text{,} \end{array}$$ (3)

where ${\overset{―}{x}}_{j}=$ mean of the j-th indicator; ${\text{s}}_{j}=$ standard deviation of the j-th indicator; *n* = number of treatments; and *m* = number of indicators.

#### Correlation matrix and eigenvalues.

The correlation matrix *R* was computed using Equation (4) as follows:


$$\begin{array}{c} \text{R}=\frac{\text{1}}{\text{n}-1}X^{\prime ⊤}X^{\prime }\text{.} \end{array}$$ (4)

Eigenvalues $\lambda_{\text{k}}\left(k=1,2,\ldots ,m\right)$ and corresponding eigenvectors were solved from |R-λI|=0.

#### Variance contribution and principal component extraction.

The variance contribution rate of the *k*-th principal component was computed using Equation (5) as follows:


$$\begin{array}{c} \eta_{\text{k}}=\frac{\lambda_{k}}{\sum_{k=1}^{m}\lambda_{k}}\times 100\%\text{.} \end{array}$$ (5)

The cumulative variance contribution rate was calculated using Equation (6) as follows:


$$\begin{array}{c} \sum_{k=1}^{\text{p}}\eta_{k}\geq 85\%, \end{array}$$ (6)

where p= number of principal components retained.

#### Comprehensive score of PCA.

The comprehensive score of PCA was calculated using Equation (7) as follows:


$$\begin{array}{c} {\text{F}}_{\text{PCA}}=\sum_{k=1}^{\text{p}}\left(\eta_{k}\cdot f_{k}\right), \end{array}$$ (7)

where ${\text{f}}_{k}=$ score of the *k*-th principal component.

#### Entropy-weighted TOPSIS method.

Entropy-weighted TOPSIS was used to compute the comprehensive performance score and rank all treatments.

#### Indicator standardization.

Positive indicators (higher is better) were calculated using Equation (8) as follows:


$$\begin{array}{c} {\text{y}}_{ij}=\frac{x_{ij}-min\left(x_{j}\right)}{max\left(x_{j}\right)-min\left(x_{j}\right)}\text{.} \end{array}$$ (8)

Negative indicators (lower is better) were calculated using Equation (9) as follows:


$$\begin{array}{c} {\text{y}}_{\text{ij}}=\frac{max\left(x_{j}\right)-x_{ij}}{max\left(x_{j}\right)-min\left(x_{j}\right)}\text{.} \end{array}$$ (9)

#### Calculation of entropy weight.

The proportion of the *i*-th treatment under the *j*-th indicator was calculated using Equation (10) as follows:


$$\begin{array}{c} {\text{p}}_{ij}=\frac{y_{ij}}{\sum_{i=1}^{n}y_{ij}}(y_{ij}>0). \end{array}$$ (10)

The entropy value of the j− th indicator was calculated using Equation (11) as follows:


$$\begin{array}{c} {\text{e}}_{j}=-k\sum_{i=1}^{n}p_{ij}ln\left(p_{ij}\right),\text{k}=\frac{\text{1}}{ln\left(n\right)}\text{.} \end{array}$$ (11)

The divergence coefficient was calculated using Equation (12) as follows:


$$\begin{array}{c} {\text{g}}_{j}=1-e_{j}\text{.} \end{array}$$ (12)

Entropy weight was calculated using Equation (13) as follows:


$$\begin{array}{c} {\text{w}}_{j}=\frac{g_{j}}{\sum_{j=1}^{m}g_{j}},\sum_{j=1}^{\text{m}}w_{j}=1\text{.} \end{array}$$ (13)

#### Weighted normalized matrix.

The weighted normalized matrix was computed using Equation (14) as follows:


$$\begin{array}{c} {\text{z}}_{ij}=w_{j}\times y_{ij}\text{.} \end{array}$$ (14)

**Positive and** n**egative** i**deal** s**olutions** were computed using Equations (15) and (16), respectively, as follows:


$$\begin{array}{c} {\text{Z}}_{\text{j}}^{+}=max\left(z_{1j},z_{2j},\ldots ,z_{nj}\right)\text{,\textasciitilde{}and} \end{array}$$ (15)

$$\begin{array}{c} {\text{Z}}_{\text{j}}^{-}=min\left(z_{1j},z_{2j},\ldots \text{,}{\text{z}}_{nj}\right)\text{.} \end{array}$$ (16)

Positive and negative Euclidean distances were calculated using Equations (17) and (18), respectively, as follows:


$$\begin{array}{c} {\text{D}}_{\text{i}}^{+}=\sqrt{\sum_{j=1}^{m}(z_{ij}-Z_{j}^{+}{)}^{\text{2}}}\text{,\textasciitilde{}and} \end{array}$$ (17)

$$\begin{array}{c} {\text{D}}_{\text{i}}^{-}=\sqrt{\sum_{j=1}^{m}{\left(z_{ij}-Z_{j}^{-}\right)}^{\text{2}}}\text{.} \end{array}$$ (18)

**Comprehensive score (closeness coefficient)** was calculated using Equation (19) as follows:


$$\begin{array}{c} {\text{C}}_{\text{i}}=\frac{D_{i}^{-}}{D_{i}^{+}+D_{i}^{-}}\text{.} \end{array}$$ (19)

Treatments were ranked in the descending order of ${\text{C}}_{\text{i}}$; the highest ${\text{C}}_{\text{i}}$ represents the optimal water–fertilizer combination.

## Results

### Effects of different treatments on the photosynthetic parameters of tomatoes

The effects of the different treatments on the photosynthetic parameters of spring-season protected tomato are shown in Table 3. Although transpiration rate and net photosynthetic rate showed no significant differences among treatments, significant variations were observed in intercellular CO₂ concentration and stomatal conductance. The intercellular CO₂ concentration of T9 (O2L1N3) was the highest, reaching 317.18 µmol∙m ⁻ ² ∙ s ⁻ ¹, significantly differing from T3, T1, and T8. Stomatal conductance was highest in T12 (O2L2N3) and lowest in T3 (O1L1N3). Statistical analysis revealed that the O × N interaction significantly impacted intercellular CO₂ concentrations and stomatal conductance (*P* < 0.05), whereas all other interactions exerted no significant effects.

**Table 3:** Effects of different treatments on tomato photosynthetic parameters.

Treatment		Transpiration rate 〔mmol/(m²·s)〕	Net photosynthetic rate 〔µmol/(m²·s)〕	Intercellular CO_2_ concentrations 〔µmol/mol〕	Stomatal conductance 〔mol/(m²·s)〕
**T1**		6.86 ± 0.52a	10.30 ± 2.59a	314.87 ± 20.47ab	0.15 ± 0.02ab
**T2**		6.20 ± 0.61a	10.22 ± 1.21a	302.89 ± 12.17abc	0.13 ± 0.02ab
**T3**		5.81 ± 1.74a	9.61 ± 1.73a	291.98 ± 29.46c	0.12 ± 0.04b
**T4**		6.11 ± 0.59a	9.93 ± 0.86a	306.86 ± 5.00abc	0.13 ± 0.01ab
**T5**		6.37 ± 0.52a	10.44 ± 0.76a	307.23 ± 6.03abc	0.14 ± 0.01ab
**T6**		5.80 ± 0.57a	10.20 ± 1.35a	297.32 ± 12.39abc	0.12 ± 0.01b
**T7**		6.07 ± 0.43a	9.54 ± 0.71a	310.45 ± 8.27abc	0.13 ± 0.01ab
**T8**		5.94 ± 0.77a	10.96 ± 0.95a	293.06 ± 14.65bc	0.12 ± 0.02ab
**T9**		6.67 ± 0.44a	9.92 ± 1.28a	317.18 ± 11.96a	0.15 ± 0.01ab
**T10**		6.10 ± 0.45a	10.45 ± 1.21a	310.46 ± 11.69abc	0.15 ± 0.02ab
**T11**		5.92 ± 0.42a	10.44 ± 0.38a	305.31 ± 10.32abc	0.13 ± 0.01ab
**T12**		6.52 ± 0.68a	10.66 ± 1.27a	314.36 ± 19.34abc	0.15 ± 0.03a
**O**	**O1**	6.19 ± 0.88a	10.11 ± 1.43a	303.53 ± 16.84a	0.13 ± 0.02a
	**O2**	6.20 ± 0.57a	10.30 ± 1.04a	309.00 ± 14.07a	0.14 ± 0.02a
**N**	**N1**	6.28 ± 0.58a	10.05 ± 1.45a	310.66 ± 12.04a	0.14 ± 0.02a
	**N2**	6.10 ± 0.57a	10.49 ± 0.84a	302.60 ± 11.34a	0.13 ± 0.02a
	**N3**	6.20 ± 1.00a	10.09 ± 1.36a	305.21 ± 21.12a	0.13 ± 0.03a
**L**	**L1**	6.26 ± 0.89a	10.06 ± 1.49a	305.49 ± 18.93a	0.13 ± 0.03a
	**L2**	6.13 ± 0.56a	10.35 ± 0.96a	306.92 ± 11.95a	0.14 ± 0.02a
**P**	**O × L**	0.677	0.747	0.742	0.300
	**O × N**	0.020	0.787	0.017*	0.016*
	**L × N**	0.615	0.632	0.447	0.630
	**O × N × L**	0.520	0.491	0.624	0.402

Notes: The water and fertilizer application status for each treatment is shown in Table 2. O represents the amount of organic fertilizer, N represents the amount of nitrogen fertilizer, and L represents the irrigation levels, whereas O×L, O×N, L×N, and O×N×L represent the interactions between different variables. Different small letters in the same column indicate significant differences between the treatments (*P* <0.05). “*” indicates a significant correlation at the *P* <0.05 level.

Under varying water and fertilizer input conditions, the photosynthetic parameters responded differently. Under the O1 condition with L1 irrigation, the values of the photosynthetic parameters decreased with reducing nitrogen application levels, whereas under L2, N2 exhibited optimal performance. Under the O2 condition, N3 performed most optimally across both the L1 and L2 irrigation levels. These patterns indicate that the effects of nitrogen on photosynthesis are modulated by both organic fertilizer application and irrigation levels.

### Effects of different treatments on the quality of tomatoes

Different treatments significantly impacted all tomato quality indicators, with the three-factor interaction (O × N × L) reaching extremely significant levels (*P* < 0.01) for most parameters (Table 4). T11 (O2L2N2) achieved the highest VC content (21.46 mg/100 g) and soluble solids content (5.62%), significantly outperforming other treatments across multiple comparison scenarios. Specifically, under the same O and N conditions, these values were 14.45% and 18.81% higher than those in T8, respectively. T5 (O1L2N2) showed the highest sugar–acid ratio (5.90), which was 31.4% higher than the lowest value in T3 (O1L1N3).

**Table 4:** Effects of different treatments on tomato quality indicators.

Treatment		VC content (mg/100g)	Soluble solids content (%)	Sugar content (%)	Total acid content (%)	Sugar–acid ratio
**T1**		16.94 ± 0.00de	5.40 ± 0.10b	3.21 ± 0.06a	0.58 ± 0.01b	5.53 ± 0.14b
**T2**		17.17 ± 0.39d	4.60 ± 0.12h	2.69 ± 0.02d	0.47 ± 0.00g	5.73 ± 0.03ab
**T3**		18.30 ± 0.00c	4.65 ± 0.08h	2.48 ± 0.03f	0.55 ± 0.01d	4.49 ± 0.12f
**T4**		17.17 ± 0.39d	4.75 ± 0.05gh	2.57 ± 0.02e	0.50 ± 0.00f	5.15 ± 0.04c
**T5**		17.07 ± 0.39d	4.90 ± 0.14ef	2.99 ± 0.09bc	0.51 ± 0.00ef	5.90 ± 0.27a
**T6**		16.49 ± 0.39e	5.07 ± 0.05d	3.06 ± 0.06b	0.60 ± 0.00a	5.10 ± 0.12c
**T7**		15.36 ± 0.39f	4.98 ± 0.14def	2.71 ± 0.05d	0.50 ± 0.00bcd	4.78 ± 0.10e
**T8**		18.75 ± 0.39c	4.73 ± 0.15gh	2.70 ± 0.01d	0.56 ± 0.00d	4.84 ± 0.07de
**T9**		19.42 ± 0.39b	5.02 ± 0.11de	2.67 ± 0.06d	0.57 ± 0.01bc	4.68 ± 0.16ef
**T10**		21.23 ± 0.39a	4.85 ± 0.16g	2.73 ± 0.04d	0.55 ± 0.01 cd	4.92 ± 0.17cde
**T11**		21.46 ± 0.39a	5.62 ± 0.10a	3.03 ± 0.03bc	0.60 ± 0.00a	5.03 ± 0.10 cd
**T12**		18.52 ± 0.39c	5.23 ± 0.31c	2.96 ± 0.03c	0.52 ± 0.01e	5.72 ± 0.11ab
**O**	**O1**	17.20 ± 0.62b	4.89 ± 0.29b	2.83 ± 0.28a	0.53 ± 0.04a	5.31 ± 0.49a
	**O2**	19.12 ± 2.11a	5.07 ± 0.32a	2.79 ± 0.15a	0.56 ± 0.03a	4.99 ± 0.36b
**N**	**N1**	17.73 ± 2.38a	4.99 ± 0.28a	2.80 ± 0.25a	0.55 ± 0.03a	5.09 ± 0.31a
	**N2**	18.40 ± 1.93a	4.96 ± 0.42a	2.85 ± 0.17a	0.53 ± 0.05a	5.37 ± 0.48a
	**N3**	18.35 ± 0.88a	4.99 ± 0.23a	2.79 ± 0.24a	0.56 ± 0.03a	4.99 ± 0.50a
**L**	**L1**	17.66 ± 1.40a	4.89 ± 0.30b	2.74 ± 0.23b	0.54 ± 0.03a	5.01 ± 0.47a
	**L2**	18.67 ± 2.07a	5.07 ± 0.32a	2.89 ± 0.19a	0.55 ± 0.04a	5.30 ± 0.39a
**P**	**O × L**	0.000*	0.000*	0.001*	0.29	0.001*
	**O × N**	0.000*	0.000*	0.000*	0.000*	0.000*
	**L × N**	0.000*	0.000*	0.000*	0.000*	0.000*
	**O × N × L**	0.000*	0.000*	0.000*	0.000*	0.072

Notes: The water and fertilizer application status for each treatment is shown in Table 2. O represents the amount of organic fertilizer, N represents the amount of nitrogen fertilizer, and L represents the irrigation levels, whereas O × L, O × N, L × N, and O × N × L represent the interactions between different variables. Different small letters in the same column indicate significant differences between the treatments (*P* < 0.05). “*” indicates a highly significant correlation at the *P* < 0.01 level. VC, vitamin C.

The analysis of individual factor effects revealed that the O2 treatment enhanced the VC and soluble solids contents but reduced the sugar–acid ratio compared with that in O1, the N2 treatment improved the VC content and sugar–acid ratio, and the L2 treatment enhanced all quality metrics compared with those in L1. These findings demonstrate that an optimal combination of irrigation and fertilization can help effectively improve tomato quality.

### Effects of different treatments on the yield and economic indicators of tomatoes

The O × L interaction had an extremely significant effect (*P* < 0.01) on tomato yield, WUE, and NPP, whereas the O × N and L × N interactions exerted no significant effects (Table 5). T4 O1L2N1) achieved the highest yield per plant (1.59 kg), significantly exceeding other treatments by 8.16%−87.05% under comparable conditions. In terms of water and nitrogen use efficiency, T7 (O2L1N1) showed the highest WUE, which was 129.47% higher than the lowest value in T10, while T6 recorded the highest NPP, exceeding T10 by 65.19%.

**Table 5:** Effects of different treatments on tomato yield, water use efficiency (WUE), and partial factor productivity of nitrogen fertilizer (NPP).

Treatment	Total water consumption (m^3^)	Irrigation volume (m^3^/ plant)	Yield (kg/ plant)	WUE (kg/m^3^)	NPP/(kg/kg)
**T1**	12.14	0.0963	1.07 ± 0.67bc	11.08 ± 6.93ab	0.89 ± 0.55ef
**T2**	12.14	0.0963	1.15 ± 0.28abc	11.96 ± 2.88ab	1.20 ± 0.29def
**T3**	12.14	0.0963	0.98 ± 0.41bc	10.18 ± 4.30abc	1.36 ± 0.58bcde
**T4**	17.21	0.1365	1.59 ± 0.14a	11.69 ± 1.06ab	1.33 ± 0.12cde
**T5**	17.21	0.1365	1.44 ± 0.51ab	10.55 ± 3.74ab	1.49 ± 0.53bcd
**T6**	17.21	0.1365	1.47 ± 0.11ab	10.81 ± 0.83ab	2.04 ± 0.15a
**T7**	12.27	0.0973	1.35 ± 0.17abc	13.86 ± 1.75a	1.12 ± 0.14def
**T8**	12.27	0.0973	1.04 ± 0.27bc	10.74 ± 2.78ab	1.09 ± 0.28def
**T9**	12.27	0.0973	1.32 ± 0.17abc	13.62 ± 1.75ab	1.84 ± 0.24ab
**T10**	17.80	0.1412	0.85 ± 0.34c	6.04 ± 2.41c	0.71 ± 0.28f
**T11**	17.80	0.1412	1.37 ± 0.28ab	9.68 ± 2.01abc	1.42 ± 0.29bcd
**T12**	17.80	0.1412	1.29 ± 0.2abc	9.16 ± 1.62bc	1.79 ± 0.32abc
**O**		O1	1.28 ± 0.43a	11.04 ± 3.59a	1.38 ± 0.52a
		O2	1.20 ± 0.30a	10.52 ± 3.33a	1.32 ± 0.47a
**N**		N1	1.22 ± 0.46a	10.66 ± 4.57a	1.01 ± 0.38c
		N2	1.25 ± 0.36a	10.73 ± 2.80a	1.29 ± 0.37b
		N3	1.26 ± 0.30a	10.94 ± 2.85a	1.75 ± 0.41a
**L**		L1	1.15 ± 0.36a	11.91 ± 3.78a	1.25 ± 0.46a
		L2	1.34 ± 0.36a	9.65 ± 2.69b	1.46 ± 0.51a
**P**		O × L	0.006*	0.009*	0.006*
		O × N	0.352	0.457	0.378
		L × N	0.381	0.472	0.294
		O × N × L	0.056	0.091	0.105

Notes: Different small letters in the same column indicate significant differences between the treatments (*P* < 0.05). “*” indicates a highly significant correlation at the *P* < 0.01 level.

Individual factor analysis indicated that the O1 treatment resulted in 6.67% higher yield than that in O2, the L2 treatment increased yield by 16.52% but reduced WUE by 23.42% compared with those in L1; and the N3 treatment led to the highest yield and NPP among nitrogen levels. These results highlight the importance of optimizing irrigation and organic fertilizer application to balance yield and resource use efficiency.

### Effects of different treatments on nitrogen, phosphorus, and potassium accumulation in tomatoes

**Fig 5:**
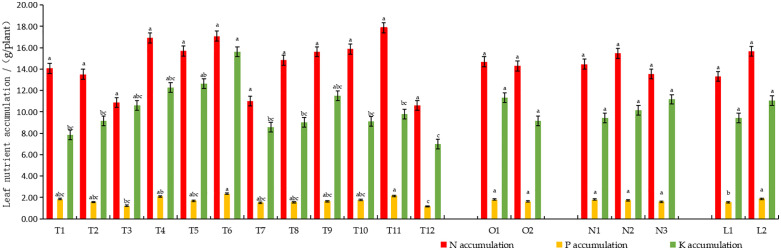
Accumulation of nitrogen, phosphorus, and potassium in leaves.

**Fig 6:**
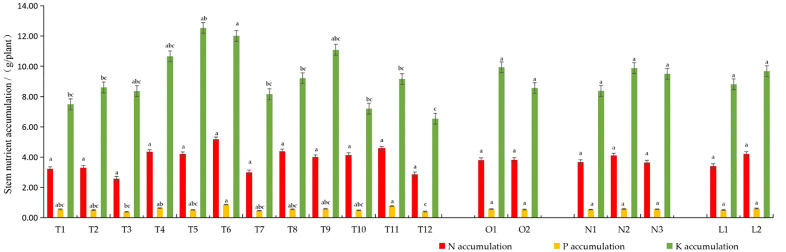
Accumulation of nitrogen, phosphorus, and potassium in stems.

**Fig 7:**
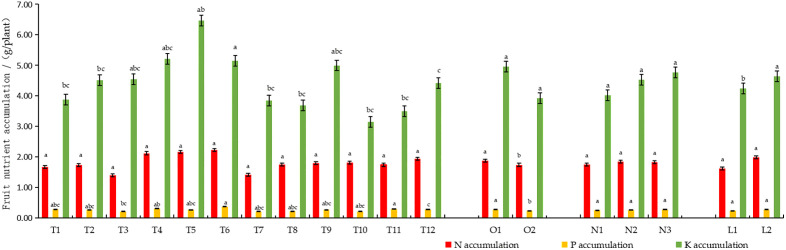
Accumulation of nitrogen, phosphorus, and potassium in fruits.

Nitrogen accumulation was significantly influenced by specific treatments and their interactions. As shown in Figs 5–7, nitrogen primarily accumulated in leaves and stems during peak growth, with the highest nitrogen accumulation levels in T11 (O2L2N2), which were 62.83% higher than those in T3 (O1L1N3), exhibiting the lowest nitrogen accumulation levels. The O × L interaction significantly affected nitrogen accumulation in stems (*P* < 0.05; Table 6), whereas the O × N and L × N interactions exerted no significant effects. Overall, the O1 and L2 treatments consistently promoted higher nitrogen accumulation levels than those in O2 and L1 across stems, leaves, and fruits.

**Table 6:** Significance levels of differences between the different interactive treatments.

Significance level of differences between different plant parts	Interactive treatment	Nitrogen accumulation	Phosphorus accumulation	Potassium accumulation
**Significance level (*P*-value) for stems**	O × L	0.021*	0.076	0.001**
	O × N	0.260	0.028*	1.000
	L × N	0.712	0.723	0.385
	O × N × L	0.041	0.002**	0.392
**Significance level (*P*-value) for leaves**	O × L	0.290	0.217	0.007**
	O × N	0.479	0.218	0.438
	L × N	0.586	0.961	0.569
	O × N × L	0.087	0.028*	0.288
**Significance level (*P*-value) for fruits**	O × L	0.053	0.245	0.001**
	O × N	0.359	0.096	0.029
	L × N	0.522	0.123	0.397
	O × N × L	0.442	0.012*	0.719

Note: “*” indicates a significant correlation at the *P* < 0.05 level, and “**” indicates a highly significant correlation at the *P* < 0.01 level.

Phosphorus accumulation patterns revealed significant interactive effects among factors. As shown in Figs 5–7, T6 (O1L2N3) achieved the highest total phosphorus content, exceeding that in T3 (O1L1N3) by 96.7%. Although phosphorus accumulation varied across the plant parts, the three-factor interaction (O × N × L) significantly impacted phosphorus accumulation in multiple organs (*P* < 0.01; Table 6). Consistent with nitrogen trends, O1 and L2 treatments generally enhanced phosphorus accumulation compared to their counterparts.

Potassium accumulation was strongly affected by water and organic fertilizer management. As shown in Figs 5–7, treatment T6 (O1L2N3) also showed the highest total potassium content, which was 82.72% higher than the lowest treatment T12 (O2L2N3). The O × L interaction exerted an extremely significant effect on potassium accumulation in stems, leaves, and fruits (*P* < 0.01; Table 6). Similar to the results obtained for the other nutrients, the O1 and L2 treatments consistently resulted in greater potassium accumulation than those in the O2 and L1 treatments.

### Effects of different treatments on the comprehensive evaluation of tomatoes

First, the evaluation indicators were divided into first-level indicators and second-level indicators. Among them, the first-level indicators included photosynthetic (F1), yield (F2), quality (F3), water use (F4), partial factor productivity of nitrogen (F5), and nitrogen–phosphorus–potassium accumulation (F6) indicators. The second-level photosynthetic indicators included transpiration rate (U1), net photosynthetic rate (U2), intercellular CO₂ concentrations (U3), and stomatal conductance (U4), whereas the quality indicators included VC content (U5), soluble solids content (U6), and sugar–acid ratio (U7).

Spearman’s correlation analysis was conducted on various indicators of tomatoes under different treatments. As shown in Table 7, among all indicators, the transpiration rate correlated significantly positively with intercellular CO₂ concentrations and stomatal conductance. Stomatal conductance was significantly positively correlated with intercellular CO₂ concentration and positively correlated with soluble solids. Sugar–acid ratio was positively correlated with water use indicators, whereas the partial factor productivity of nitrogen indicators correlated positively with the VC content and yield indicators. The relationships between data indicators showed both differences and overlapping correlations, necessitating further comprehensive evaluation and analysis of the indicators.

**Table 7:** Spearman’s correlation between different indicators.

Index	*U1*	*U2*	*U3*	*U4*	*U5*	*U6*	*U7*	*F2*	*F4*	*F5*	*F6*
***U1***	1										
***U2***	0.133	1									
***U3***	0.832**	0.042	1								
***U4***	0.832**	0.336	0.930**	1							
***U5***	−0.148	0.394	0.007	0.127	1						
***U6***	−0.266	0.126	0.531	0.601*	0.056	1					
***U7***	0.455	0.448	0.147	0.308	−0.380	0.126	1				
***F2***	−0.014	−0.273	0.014	−0.056	−0.401	0.329	0.336	1			
***F4***	0.203	−0.675*	0.133	−0.154	−0.549	−0.147	−0.105	0.343	1		
***F5***	−0.42	−0.189	−0.028	−0.028	0.014	0.322	0.084	0.594*	0.014	1	
***F6***	−0.126	0.378	−0.266	−0.224	0.176	−0.434	0.350	0.070	−0.217	0.056	1

Note(s): “*” indicates a significant correlation at the *P* < 0.05 level, and “**” indicates a highly significant correlation at the *P* < 0.01 level. *U1* is the transpiration rate, *U2* is the net photosynthetic rate, *U3* represents intercellular CO₂ concentrations, *U4* is stomatal conductance,*U5* represents the vitamin C content, *U6* represents soluble solids, *U7* is the sugar–acid ratio, *F2* represents yield indicators, *F4* represents water use indicators, *F5* is the partial factor productivity of nitrogen indicators, and *F6* represents nitrogen–phosphorus–potassium accumulation indicators.

The PCA method was used to comprehensively evaluate the photosynthetic indicators and quality indicators of tomatoes. First, the secondary indicators included in the photosynthetic indicators and quality indicators were normalized, followed by PCA.

The contribution rates of the characteristic values of tomato photosynthetic indices are shown in Table 8, and two principal components are extracted. The “variance explanation rates” of the two principal components were 68.684% and 25.867%, respectively, with a cumulative variance explanation rate of 94.552%. The principal component expressions can be represented using Equations (20) and (21) as follows:

**Table 8:** Contribution rates of the characteristic eigenvalues of tomatoes.

No.	Eigenvalue	Variance interpretation rate (%)	Cumulative variance interpretation rate (%)
**1**	2.747	68.684	68.684
**2**	1.035	25.867	94.552
**3**	0.180	4.507	99.059
**4**	0.038	0.941	100.000


$$\begin{array}{c} Y_{\text{1}}=0.565U_{\text{1}}+0.103U_{\text{2}}+0.566U_{\text{3}}+0.59U_{\text{4}}\text{,\textasciitilde{}and} \end{array}$$ (20)

$$\begin{array}{c} Y_{\text{2}}=-0.062U_{\text{1}}+0.967U_{\text{2}}-0.223U_{\text{3}}+0.103U_{\text{4}}\text{.} \end{array}$$ (21)

The factors that have the greatest impact on the first principal component and the second principal component are transpiration rate and net photosynthetic rate, respectively. Taking the first two principal components as the evaluation indicators of tomato photosynthetic indices, the comprehensive evaluation equation can be expressed using Equation (22) as follows:


$$\begin{array}{c} Y=0.726Y_{\text{1}}+0.273Y_{\text{2}}\text{.} \end{array}$$ (22)

The contribution rates of eigenvalues for tomato quality indicators are shown in Table 9. The two principal components, with variance explanation rates of 44.726% and 38.517%. respectively, and a cumulative variance explanation rate of 83.243%, were extracted. The expressions of the principal components can be represented using Equations (23) and (24) as follows:

**Table 9:** Contribution rates of the characteristic eigenvalues of tomato quality indicators.

No.	Eigenvalue	Variance interpretation rate (%)	Cumulative variance interpretation rate (%)
**1**	1.342	44.726	44.726
**2**	1.156	38.517	83.243
**3**	0.503	16.757	100.000


$$\begin{array}{c} Z_{\text{1}}=0.884U_{\text{5}}+0.353U_{\text{6}}-0.661U_{\text{7}}\text{,\textasciitilde{}and} \end{array}$$ (23)

$$\begin{array}{c} Z_{\text{2}}=0.127U_{\text{5}}+0.862U_{\text{6}}+0.63U_{\text{7}}\text{.} \end{array}$$ (24)

The components with the greatest impact on the first and second principal components were the VC and soluble solids contents, respectively. Taking the first two principal components as the evaluation indicators for tomato quality indicators, the comprehensive evaluation equation can be expressed using Equation (25) as follows:


$$\begin{array}{c} Z=0.537Z_{\text{1}}+0.463Z_{\text{2}}\text{.} \end{array}$$ (25)

Based on Equations (22) and (25), the comprehensive scores and rankings of tomato photosynthetic and quality indicators were obtained (Table 10).

**Table 10:** Scores and rankings of Tier 1 indicators.

Treatment	F1		F2		F3		F4		F5		F6	
	Number	Ranking	Number	Ranking	Number	Ranking	Number	Ranking	Number	Ranking	Number	Ranking
**T1**	1.77	2	1.07	9	0.39	5	11.08	5	1.47	4	5.35	11
**T2**	−0.38	7	1.15	8	−0.96	12	11.96	3	1.19	7	5.99	3
**T3**	−2.16	11	0.98	11	−0.49	8	10.18	9	0.81	12	5.48	9
**T4**	−0.51	8	1.59	1	−0.66	10	11.69	4	2.21	1	5.56	6
**T5**	0.58	5	1.44	3	−0.62	9	10.55	8	1.49	3	7.18	1
**T6**	−1.40	12	1.47	2	−0.10	6	10.81	6	1.22	6	5.82	4
**T7**	−0.67	9	1.35	5	−0.68	11	13.86	1	1.87	2	4.48	12
**T8**	−0.68	10	1.04	10	−0.26	7	10.74	7	1.08	10	5.71	5
**T9**	1.30	3	1.32	6	0.41	4	13.62	2	1.10	9	5.54	7
**T10**	0.59	4	0.85	12	0.61	2	6.04	12	1.18	8	6.14	2
**T11**	−0.25	6	1.37	4	1.88	1	9.68	10	1.42	5	5.49	8
**T12**	1.81	1	1.29	7	0.48	3	9.16	11	1.07	11	5.41	10

Notes: Treatment codes: T1 = O1L1N1, T2 = O1L1N2,..., and T12 = O2L2N3.

The entropy weight TOPSIS evaluation method was used to evaluate the values and rankings of the primary evaluation indicators in the tomato comprehensive evaluation system. The results are shown in Table 11, wherein T11 refers to the treatment representing the combination of L2 (85–90% field capacity), O2 (low organic fertilizer), and N2 (20% reduced nitrogen), ranked first in the comprehensive ranking.

**Table 11:** Comprehensive ranking of different treatments.

Treatment	D+	D-	C	Ranking
T1	2.484	1.144	0.315	9
T2	2.552	0.533	0.173	11
T3	2.431	0.494	0.169	12
T4	2.12	0.865	0.29	10
T5	1.876	1.177	0.385	8
T6	1.74	1.249	0.418	7
T7	1.701	1.367	0.446	6
T8	1.429	1.597	0.528	5
T9	0.926	2.044	0.688	4
T10	0.838	2.169	0.721	3
T11	0.599	2.533	0.809	1
T12	0.764	2.643	0.776	2

## Discussion

### Effects of different treatments on the photosynthetic capacity and quality of tomatoes

Nitrogen fertilizer serves as the material basis of the photosynthetic system, which needs to be controlled within an appropriate range. Organic fertilizer complements nitrogen fertilizer by improving the soil environment and regulating metabolic processes, particularly maintaining or even enhancing the photosynthetic efficiency under reduced nitrogen input conditions. Additionally, setting a reasonable lower limit for irrigation can also help promote the photosynthetic capacity of crop leaves, such as in cucumber [23]. In the present study, the interaction between O and N exerted a significant impact on the transpiration rate, intercellular CO₂ concentration, and stomatal conductance of tomatoes,This is mainly because gradual mineralization of organic fertilizer continuously improves soil nitrogen availability and crop nutrient acquisition; sustained variation in leaf nitrogen status modulates stomatal traits, resulting in significant O × N interactive effects on Ci and Gs. Prior research shows photosynthetic parameters diverge sharply across treatments right after fertigation and rewatering due to unequal water and nutrient inputs. As plants mature, previously stressed tomato plants restore root nutrient uptake and stomatal function, gradually minimizing intertreatment variation and aligning overall photosynthetic performance across experimental groups [24], In the present experiment, most treatments displayed nonsignificant differences in photosynthetic metrics apart from a small subset. This pattern likely stems from inconsistent timing during leaf gas-exchange measurements, where multifactor coupling homogenizes leaf photosynthetic capacity. This observation aligns with earlier published findings.Moreover, the non-significant effects of the O × N interaction on photosynthetic parameters may stem from the compensatory role of organic fertilizer in enhancing nitrogen use efficiency and stabilizing the photosynthetic apparatus, thereby mitigating the effects of nitrogen fluctuation.

Setting the lower limit of water irrigation affects the irrigation amount during the tomato growth cycle, while the rational combination of irrigation, organic fertilizer, and nitrogen fertilizer plays a crucial role in improving tomato quality. Existing studies have shown that treatments such as irrigation and fertilization significantly influence the sugar-acid ratio, organic acids, soluble sugars, and lycopene content in tomatoes. Setting a reasonable lower irrigation limit and reducing nitrogen with combined organic fertilizer can enhance tomato quality indicators [25]. In the present study, T11 had the highest VC and soluble solids contents, whereas T5 exhibited the highest sugar–acid ratio, indicating that setting a higher value for the lower irrigation limit and reducing nitrogen input levels by 20% can help achieve superior quality performance. The O × L, O × N, L × N, and O × N × L interactions significantly affected the VC content, soluble solids content, and sugar content of tomatoes; however, the interactive relationships require further experimental verification. The water–fertilizer coupling effect likely regulates tomato quality by modulating root water and nutrient uptake, impacting photoassimilate distribution and metabolic substrate supply. This mechanism deserves further investigation under different water–organic–nitrogen management strategies.

### Effects of different treatments on yield, economic indicators, and nutrient accumulation

The results obtained in this study revealed that setting the lower limit of irrigation initiation and the combined application of organic and nitrogen fertilizers improved tomato yields and economic indicators to a certain degree. Studies have shown that tomato yield increases with the increase of lower irrigation limit, reaching the maximum when the lower limit is 80% of soil water content, while deficit irrigation leads to yield reduction [26,27]. Combined application of organic fertilizer can increase dry matter and fresh fruit yield, and nitrogen fertilizer application can improve tomato yield and water-fertilizer productivity [28,29]. In this experiment, a higher irrigation lower limit increased the total irrigation amount and average yield but reduced WUE. Application of higher amounts of organic fertilizers improved yield, WUE, and NPP, whereas a moderate reduction in nitrogen inputs increased yield and WUE, although excessive reduction decreased NPP. These findings generally align with existing research.

In this study, the O × L interaction had extremely significant effects on tomato yield, WUE, and NPP (*P* < 0.01), whereas the O × N and L × N interactions had no significant effects on the three indicators. However, some studies have shown that the combined application of organic and nitrogen fertilizers, as well as water–nitrogen coupling, can significantly affect tomato yield and water–fertilizer utilization rate [30], which contrasts with the results of the present study. This discrepancy may be explained by the integrated three-factor design potentially masking binary interaction effects. Furthermore, the response of the soil–plant system to water–fertilizer coupling depends heavily on specific conditions, such as soil texture, organic matter content, and microbial activity, which may vary across studies. The three-factor interaction (O × N × L) exhibited non-significant effects on yield and WUE but significant effects on NPP, requiring further experimental validation.

Maintaining a certain level of accumulation of nitrogen, phosphorus, and potassium during the tomato growth period can help promote plant growth and development, playing a vital role in improving fruit yield and quality. Studies have shown that irrigation amount has a greater impact on nitrogen accumulation in tomato fruits and leaves than on stem and root biomass [31]. In the present study, treatment L2, which obtained a higher irrigation amount, resulted in higher levels of nitrogen, phosphorus, and potassium in fruits, stems, and leaves than those in treatment L1. However, the O × L and O × N × L interactions related to irrigation significantly affected nitrogen accumulation in stems, with no significant effects on nitrogen accumulation in fruits and leaves, which differs from the results of other relevant studies and may be related to the soil water retention capacity and root distribution patterns under combined water–organic management, which could alter nutrient mobility and plant uptake preferences among organs.

Other studies have indicated that nitrogen application has no significant effects on phosphorus and potassium accumulation, whereas a combined application of nitrogen fertilizers in different forms can help improve the accumulation of nitrogen, phosphorus, and potassium in tomatoes. Under the same nitrogen application rate, drip irrigation fertilization with organic fertilizers has the same good fertilizer efficiency as that with mineral fertilizers, while ensuring higher environmental sustainability [32, 33]. In the present study, the two-factor interactions related to nitrogen application significantly affected only the phosphorus content in stems, with no significant effects on phosphorus and potassium accumulation in fruits and leaves. Nitrogen reduction helped improve the overall accumulation of nitrogen, phosphorus, and potassium in fruits, stems, and leaves. Treatment O1 resulted in increased total amounts of nitrogen, phosphorus, and potassium in fruits, stems, and leaves compared with those in treatment O2, which is consistent with the relevant research conclusions, suggesting that organic amendments enhance nutrient synergy in the soil–plant system, likely by improving rhizosphere ecology and promoting microbial-mediated nutrient transformation and availability.

## Conclusions

The study found that the interaction between O × N has a significant impact on the transpiration rate, intercellular CO₂ concentration, and stomatal conductance of tomatoes. The interaction between O × L has an extremely significant impact on tomato yield, WUE, NPP, and potassium accumulation in stems, leaves, and fruits (*P* < 0.01). The O × N × L interaction had extremely significant effects on the contents of VC, total soluble solids, and soluble sugars, as well as on the total acidity and phosphorus accumulation in the stems, leaves, and fruits of greenhouse tomatoes. To maximize the comprehensive benefits of tomato growth, based on scientific soil testing, it is recommended that the lower irrigation limit and fertilizer application scheme for Spring-season protected tomatoes in this region are as follows: applying a lower level of organic fertilizer, reducing nitrogen fertilizer application by 20%, and setting the upper limit of irrigation at 85–90% FC.

## Supporting information

S1 Fig Tomato growth stages. (TIF)

## References

[1] Mendonça SR, Ávila MCR, Vital RG, Evangelista ZR, Pontes N de C, Nascimento A dos R. The effect of different mulching on tomato development and yield. Scientia Horticulturae. 2021;275:109657. doi: 10.1016/j.scienta.2020.109657

[2] Li J, Xiang Z, Wang X, Guo Y, Huang Z, Liu L. Current situation and prospect of tomato industry in China during the “13th five-year plan” period. China Vegetables. 2021;2:13–20. doi: 10.19928/j.cnki.1000-6346.2021.2009

[3] Xu J, Xiang Z, Liu M. Analysis of cost-benefit and research on paths for quality improvement and efficiency enhancement in China’s tomato industry. China Vegetables. 2025;6:1–8. doi: 10.19928/j.cnki.1000-6346.2025.1033

[4] Hochmuth G, Hanlon E. Summary of N, P, and K Research with Tomato in Florida. EDIS. 2011;2011(10). doi: 10.32473/edis-cv236-2011

[5] Huang Y, Yang Y-R, Yu J-X, Huang J-X, Kang Y-F, Du Y-R, et al. Interaction of the Coupled Effects of Irrigation Mode and Nitrogen Fertilizer Format on Tomato Production. Water. 2023;15(8):1546. doi: 10.3390/w15081546

[6] Wang X, Xing Y. Evaluation of the effects of irrigation and fertilization on tomato fruit yield and quality: a principal component analysis. Sci Rep. 2017;7(1):350. doi: 10.1038/s41598-017-00373-8 28336916 PMC5428234

[7] Barickman TC, Simpson CR, Sams CE. Waterlogging Causes Early Modification in the Physiological Performance, Carotenoids, Chlorophylls, Proline, and Soluble Sugars of Cucumber Plants. Plants (Basel). 2019;8(6):160. doi: 10.3390/plants8060160 31181725 PMC6630288

[8] Leghari SJ, Han W, Soomro AA, Shoukat MR, Zain M, Wei Y, et al. Navigating water and nitrogen practices for sustainable wheat production by model-based optimization management systems: A case study of China and Pakistan. Agricultural Water Management. 2024;300:108917. doi: 10.1016/j.agwat.2024.108917

[9] Wang Q, Jia Y, Pang Z, Zhou J, Scriber KE II, Liang B, et al. Intelligent fertigation improves tomato yield and quality and water and nutrient use efficiency in solar greenhouse production. Agricultural Water Management. 2024;298:108873. doi: 10.1016/j.agwat.2024.108873

[10] Leghari SJ, Han W, Hu K, Laghari Y, Wei Y, Cui L. What should we do for water security? A technical review on more yield per water drop. J Environ Manage. 2024;370:122832. doi: 10.1016/j.jenvman.2024.122832 39396484

[11] Shu L-Z, Liu R, Min W, Wang Y, Hong-mei Y, Zhu P, et al. Regulation of soil water threshold on tomato plant growth and fruit quality under alternate partial root-zone drip irrigation. Agricultural Water Management. 2020;238:106200. doi: 10.1016/j.agwat.2020.106200

[12] Wang Q, Jia Y, Pang Z, Zhou J, Scriber KE II, Liang B, et al. Intelligent fertigation improves tomato yield and quality and water and nutrient use efficiency in solar greenhouse production. Agricultural Water Management. 2024;298:108873. doi: 10.1016/j.agwat.2024.108873

[13] Hasnain M, Chen J, Ahmed N, Memon S, Wang L, Wang Y. The Effects of Fertilizer Type and Application Time on Soil Properties, Plant Traits, Yield and Quality of Tomato. Sustainability. 2020;12:9065. doi: 10.3390/su12219065

[14] Wu Y, Yan S, Fan J, Zhang F, Zheng J, Guo J, et al. Combined application of soluble organic and chemical fertilizers in drip fertigation improves nitrogen use efficiency and enhances tomato yield and quality. J Sci Food Agric. 2020;100(15):5422–33. doi: 10.1002/jsfa.10593 32564361

[15] Wu Y, Yan S, Fan J, Zhang F, Zheng J, Guo J, et al. Combined application of soluble organic and chemical fertilizers in drip fertigation improves nitrogen use efficiency and enhances tomato yield and quality. J Sci Food Agric. 2020;100(15):5422–33. doi: 10.1002/jsfa.10593 32564361

[16] Fan M, Qin Y, Jiang X, Cui N, Wang Y, Zhang Y, et al. Proper Deficit Nitrogen Application and Irrigation of Tomato Can Obtain a Higher Fruit Quality and Improve Cultivation Profit. Agronomy. 2022;12(10):2578. doi: 10.3390/agronomy12102578

[17] Zhang Z, Yu Z, Zhang Y, Shi Y. Finding the fertilization optimization to balance grain yield and soil greenhouse gas emissions under water-saving irrigation. Soil and Tillage Research. 2021;214:105167. doi: 10.1016/j.still.2021.105167

[18] Piri H, Naserin A, Albalasmeh AA. Interactive effects of deficit irrigation and vermicompost on yield, quality, and irrigation water use efficiency of greenhouse cucumber. J Arid Land. 2022;14(11):1274–92. doi: 10.1007/s40333-022-0035-7

[19] Zhou C, Zhang H, Yu S, Chen X, Li F, Wang Y, et al. Optimizing water and nitrogen management strategies to improve their use efficiency, eggplant yield and fruit quality. Front Plant Sci. 2023;14:1211122. doi: 10.3389/fpls.2023.1211122 37767295 PMC10519791

[20] Wang C, Gu F, Chen J, Yang H, Jiang J, Du T, et al. Assessing the response of yield and comprehensive fruit quality of tomato grown in greenhouse to deficit irrigation and nitrogen application strategies. Agricultural Water Management. 2015;161:9–19. doi: 10.1016/j.agwat.2015.07.010

[21] Zhu K, Zhao Y, Ma Y, Zhang Q, Kang Z, Hu X. Drip irrigation strategy for tomatoes grown in greenhouse on the basis of fuzzy Borda and K-means analysis method. Agricultural Water Management. 2022;267:107598. doi: 10.1016/j.agwat.2022.107598

[22] Qu F, Zhang Q, Jiang Z, Zhang C, Zhang Z, Hu X. Optimizing irrigation and fertilization frequency for greenhouse cucumber grown at different air temperatures using a comprehensive evaluation model. Agricultural Water Management. 2022;273:107876. doi: 10.1016/j.agwat.2022.107876

[23] Dong H, Li F, Xuan X, Ahiakpa JK, Tao J, Zhang X, et al. The genetic basis and improvement of photosynthesis in tomato. Horticultural Plant Journal. 2025;11(1):69–84. doi: 10.1016/j.hpj.2023.06.007

[24] Peco JD, Centeno A, Moratiel R, Villena J, López-Perales JA, Moreno MM, et al. Intermittent versus continuous drought: chlorophyll a fluorescence reveals photosystem resilience in tomato. Front Plant Sci. 2025;16:1699777. doi: 10.3389/fpls.2025.1699777 41368144 PMC12683661

[25] Li B, Bao Z-R, Yao M-Z, Li C-X, Sun X-L. Effects of irrigation lower limit and straw returning amount on yield, quality and water use efficiency of greenhouse tomato. Ying Yong Sheng Tai Xue Bao. 2020;31(2):493–500. doi: 10.13287/j.1001-9332.202002.030 32476342

[26] Sun Y, Duan L, Zhong H, Cai H, Xu J, Li Z. Effects of irrigation-fertilization-aeration coupling on yield and quality of greenhouse tomatoes. Agricultural Water Management. 2024;299:108893. doi: 10.1016/j.agwat.2024.108893

[27] Shabbir A, Mao H, Ullah I, Buttar NA, Ajmal M, Lakhiar IA. Effects of Drip Irrigation Emitter Density with Various Irrigation Levels on Physiological Parameters, Root, Yield, and Quality of Cherry Tomato. Agronomy. 2020;10(11):1685. doi: 10.3390/agronomy10111685

[28] Togun AO, Akanbi WB. Comparative Effectiveness of Organic-Based Fertilizer To Mineral Fertilizer on Tomato Growth and Fruit Yield. Compost Science & Utilization. 2003;11(4):337–42. doi: 10.1080/1065657x.2003.10702143

[29] Li Y, Sun Y, Liao S, Zou G, Zhao T, Chen Y, et al. Effects of two slow-release nitrogen fertilizers and irrigation on yield, quality, and water-fertilizer productivity of greenhouse tomato. Agricultural Water Management. 2017;186:139–46. doi: 10.1016/j.agwat.2017.02.006

[30] Tao Y, Liu T, Wu J, Wu Z, Liao D, Shah F, et al. Effect of Combined Application of Chicken Manure and Inorganic Nitrogen Fertilizer on Yield and Quality of Cherry Tomato. Agronomy. 2022;12(7):1574. doi: 10.3390/agronomy12071574

[31] Ayankojo IT, Morgan KT, Ozores-Hampton M, Migliaccio KW. Effects of Real-time Location-specific Drip Irrigation Scheduling on Water Use, Plant Growth, Nutrient Accumulation, and Yield of Florida Fresh-market Tomato. horts. 2018;53(9):1372–8. doi: 10.21273/hortsci13183-18

[32] Wang C, Wu G, Wang H, Wang J, Yuan M, Guo X, et al. Optimizing Tomato Cultivation: Impact of Ammonium–Nitrate Ratios on Growth, Nutrient Uptake, and Fertilizer Utilization. Sustainability. 2024;16(13):5373. doi: 10.3390/su16135373

[33] Farneselli M, Tosti G, Onofri A, Benincasa P, Guiducci M, Pannacci E, et al. Effects of N sources and management strategies on crop growth, yield and potential N leaching in processing tomato. European Journal of Agronomy. 2018;98:46–54. doi: 10.1016/j.eja.2018.04.006

