# Supplementary material for: Effects of different irrigation lower limits and combined application of reduced nitrogen with organic fertilizer on spring-season tomatoes (Solanum lycopersicum)
Source: PLoS One. 2026 Aug 11;21(8):e0354927. doi: 10.1371/journal.pone.0354927 (PMC13460604; doi:10.1371/journal.pone.0354927)
Supplement: Supplementary file 1 [file pone.0354927.s007.pdf]

Response to Reviewers

Point.1: The authors measured soil field capacity, but the specific values are not provided in the text. Please supplement this information.

Response 1: Thank you very much for your suggestion.Field capacity data have been added to the manuscript.

Point.2: In Table 1, the soil type is given as loam, but the specific particle size distribution is not provided. Please provide this information.

Response 2: Thank you very much for your suggestion. The soil particle size data have been supplemented.

Point.3: The basis for the organic fertilizer application rates is missing. Please provide the rationale for the chosen rates.

Response 3: Thank you very much for your suggestion.Fertilization was performed in accordance with the Chinese agricultural industry standard Guidance for the Fertilization Control Techniques of Protected Vegetables (NY/T 3832-2021). The grading criteria for soil fertility specified in this standard are as follows:

Item	Low fertility	Medium fertility	High fertility	Extremely high fertility
Organic matter (g/kg)	＜20	≥20，＜30	≥30，＜40	≥40
Available nitrogen (mg/kg)	＜50	≥50，＜100	≥100，＜150	≥150
Available phosphorus (mg/kg)	＜50	≥50，＜100	≥100，＜150	≥150
Available potassium (mg/kg)	＜150	≥150，＜200	≥200，＜300	≥300

According to the soil test data in Table 1, the soil organic matter content corresponds to low fertility. Fertilizer application rates of N, P and K were determined

referring to the above standard following the same calculation principle. Related explanations have been supplemented in the manuscript.

Point.4: In Equation (1), "total" should be written as a subscript.

Response 4: Thank you very much for your suggestion.The word "total" in Equation (1) has been changed to subscript form.

Point.5: Although transpiration rate and net photosynthetic rate showed no significant differences among treatments, intercellular CO_2 concentration and stomatal conductance did show significant differences. Please examine whether this has been explained in the Discussion section.

Response 5: Thank you very much for your suggestion.We have added corresponding explanatory content in the Discussion section.

Point.6: Lines 374–377 state: *"However, except for a few treatments, there were no obvious differences in photosynthetic indices between different treatments, which may be attributed to the imprecise timing of photosynthetic measurements, leading to convergent leaf photosynthetic capacity under multi-factor coupling."* Attributing the lack of differences to measurement timing seems somewhat tenuous. Is there any literature to support this statement?

Response 6: Thank you very much for your suggestion.Relevant explanations have been supplemented in the manuscript, and literatures supporting these conclusions have been cited in the reference list.

Point.7: In the Conclusions, the phrase *"setting the soil irrigation level at 85% FC to 90% FC"* is not accurate. Should it instead read as setting the **upper limit** of irrigation at 85–90% FC?

Response 7: Thank you very much for your suggestion.This expression has been revised to “setting the upper limit of irrigation at 85–90% FC” in the Conclusions as suggested.
