# Supplementary material for: Effects of different irrigation lower limits and combined application of reduced nitrogen with organic fertilizer on spring-season tomatoes (Solanum lycopersicum)
Source: PLoS One. 2026 Aug 11;21(8):e0354927. doi: 10.1371/journal.pone.0354927 (PMC13460604; doi:10.1371/journal.pone.0354927)
Supplement: Supplementary file 2 [file pone.0354927.s002.docx]

**The effects of different irrigation lower limits and combined application of reduced nitrogen with organic fertilizer on spring-season tomato**

**Reviewer comments**

This study systematically explored the effects of three factors (irrigation minimum, organic fertilizer application rate, and nitrogen fertilizer reduction) on the photosynthetic characteristics, yield, quality, and water and nitrogen use efficiency of greenhouse spring tomatoes through a crossover experiment. Principal component analysis and the entropy-weighted TOPSIS method were used for comprehensive evaluation. The study design was sound, the analytical methods were appropriate, and it offers practical guidance, particularly in the area of water-fertilizer coordination. However, the manuscript requires further improvement in terms of experimental design, interpretation of results, and language .

1. Materials and Methods: Many essential details are missing, and in its current form, the reader cannot replicate the experiments. In fact, it is even difficult to determine whether the methodology adequately addresses the study objectives. The description of the greenhouse is vague and lacks key details about environmental conditions (e.g., temperature control, ventilation, and light transmittance), which are crucial for reproducibility.

2. High clay content in the soil affects water movement and nutrient availability, but this is not mentioned in the basic properties of soil.

3. In line 98, the author states that “the growth period was 102 days”， is there any basis for this statement? What signifies the end of the experiment?

4. In line 156 , the author mentions the definition of WUE, but uses WUEy in the formula. Does it have the same meaning? I hope the author will correct this.

5. The Results section reiterates data already presented in the tables, failing to highlight key findings. I recommend that the authors streamline the description and prioritize significant differences and interaction effects.

6. The current discussion focuses primarily on describing the results, lacking in-depth comparisons with existing studies and mechanistic explanations. The authors are advised to provide more explanations for any discrepancies between the results and the literature (e.g., the possible reasons for the non-significant interaction between ON and LN) and to explore the mechanisms of water-fertilizer coupling in the soil-plant system.

7. The language needs further polishing. The“lower limit of irrigation”should be unified as “irrigation threshold” or “lower limit of irrigation”. It is recommended that a native English speaker polish the entire text to ensure accurate terminology and fluent sentences.

8. The reference format is not uniform, please correct it.
