# Supplementary material for: Effects of different irrigation lower limits and combined application of reduced nitrogen with organic fertilizer on spring-season tomatoes (Solanum lycopersicum)
Source: PLoS One. 2026 Aug 11;21(8):e0354927. doi: 10.1371/journal.pone.0354927 (PMC13460604; doi:10.1371/journal.pone.0354927)
Supplement: Supplementary file 3 [file pone.0354927.s003.docx]

Response to Reviewer 1comments

**Point.1:** The effects of different irrigation lower limits and combined application ofreduced nitrogen with organic fertilizer on spring-season tomato.Improve the title OR just remove ‘’The’’. Effects of different irrigation lower limits and combined application of reduced nitrogen with organic fertilizer on spring-season tomato.

Response 1: Thank you very much for your suggestion. The revised title "Greenhouse tomato is a high-value cash crop globally, but inefficient water and nitrogen management in greenhouse systems often causes resource waste, environmental risks, and reduced yield/quality—key challenges for sustainable cultivation." is more concise and in line with the academic expression norms of journal titles, and we have updated it in the manuscript accordingly.

**Point.2:** haijie li check the author name. First characters should be capitalized.

Response 2: Thank you very much for your question. We have revised the author name to "Haijie Li" in accordance with your suggestion that the first characters of both the given name and surname should be capitalized.

**Point.3:** In abstract, write one to two sentences of context or the importance of the study.

This is too long.

Response 3:Thank you very much for your question.We have supplemented one background sentence at the beginning of the abstract to clarify the research significance: “Greenhouse tomato is a key cash crop in many regions, but irrational water and fertilizer management often leads to low resource use efficiency and compromised yield/quality.” This sentence highlights the practical problem addressed by the study, making the research purpose more targeted.

**Point.4:** What was the irrigation amount (mm) in each treatment? Write irrigation amount for irrigation levels 75%FC～90%FC (L1)and 85%FC～90%FC (L2).

Response 4:Thank you very much for your question.We set the irrigation amounts based on soil moisture content measured by soil moisture sensors, with reference to soil field capacity. Due to the spatial heterogeneity of soil, the field capacity varies across different locations, resulting in differences in the actual irrigation amounts among treatments even under the same field capacity-based settings. The specific irrigation amounts for each treatment are detailed in Table 5. Therefore, we have revised the text by adding "where FC refers to soil field capacity" after the sentence "Two irrigation levels were set: 75%FC～90%FC (L1) and 85%FC～90%FC (L2)" to facilitate readers' understanding.

**Point.5:** When was the experiment conducted? Is it one year or two year or more?

Response 5:Thank you for your question regarding the experimental timeline. We have incorporated the relevant information into the abstract to clarify the duration and timing of the experiment.

The experiment was conducted in 2024, with the tomatoes planted on March 14, the formal experiment initiation on April 1, a total growth period of 102 days, and vine pulling completed on June 24. This specifies that the study was based on a one-year field trial, as detailed in the revised abstract.

**Point.6:** English writing should be improved in the abstract; it needs to be rewritten.For topdressing, nitrate nitrogen was applied with three nitrogen levels: conventional nitrogen rate (N1, 352.14 kg·hm⁻²), 20% reduced nitrogen (N2, 281.72 kg·hm⁻²), and 40% reduced nitrogen (N3, 211.29 kg·hm⁻²). A three-factor cross experiment was conducted.

The whole abstract is composed of the materials and methods section. The context is missing and the results are not detailed. The results showed that applying a lower level of organic fertilizer, reducing nitrogen application by 20%, and setting the irrigation level at 85%FC～90%FC could maximize the comprehensive benefits of tomato growth.

Response 6:Thank you for your detailed comments on the abstract. We fully agree with your suggestions to improve the English expression, balance the structure, and enrich the results. We have revised the abstract accordingly:

Enhanced English writing: We refined awkward phrases (e.g., revised "For topdressing, nitrate nitrogen was applied with three nitrogen levels" to "three topdressing nitrate nitrogen levels") .

Strengthened context: We added background on sustainable cultivation challenges (resource waste, environmental risks) to clarify the study’s significance, addressing the "missing context" concern.

Detailed results: We replaced the vague description of "comprehensive benefits" with specific findings of treatment T11, including its highest values for vitamin C (21.46 mg/100g) and soluble solids content (5.62%), as well as the highest total nitrogen content in fruits, stems, and leaves (62.83% higher than the lowest treatment).

We clarified the application of principal component analysis (for photosynthetic and quality indicators) and the entropy-weighted TOPSIS method, explicitly listing the first-level evaluation indicators (F1–F6) and confirming that T11 ranked first in the comprehensive ranking

**Point.7:** The research gap should be better emphasized. Avoid unnecessary wording and sentences. Completed the introduction section in threeparagraphs. The longer the introduction section higher the mistakes and the lower the writing efficiency.

Response 7:Thank you very much for your question.We have restructured the introduction into 3 concise paragraphs, removed redundant content (e.g., redundant descriptions of greenhouse advantages, repetitive mentions of fertilizer types), and explicitly highlighted 3 core research gaps:

Regional applicability gap: Existing irrigation strategies (e.g., 70% FC in Huaibei, 80–85% FC in Shouguang) lack verification in Shijiazhuang with distinct soil-climatic conditions;

Interaction gap: Optimal ratio of organic-to-inorganic fertilizers and its coupling with irrigation remain unclear;

Evaluation method gap: Single indices are insufficient, requiring integrated methods (PCA, entropy-weighted TOPSIS) for comprehensive assessment.

This revision improves writing efficiency and reduces potential errors by streamlining logic and removing non-essential details.

**Point.8:** Avoid repetition of study area ‘’Shandong, Hebei, and Henan’’, Shijiazhuang, Hebei.

Response 8:Thank you very much for your question.We have optimized the mention of study areas to avoid repetition:

In Paragraph 1, we only note "Shandong, Hebei, and Henan as core planting provinces" to clarify China’s tomato production layout, without redundant elaboration;

In Paragraph 2, we contrast existing research regions (Huaibei, Shouguang) with the target region (Shijiazhuang) only once, focusing on the "regional applicability gap" rather than repeating "Hebei" separately;

No further redundant mentions of these regions appear in Paragraph 3.

This ensures clarity of regional context while eliminating repetitive content.

**Point.9:** Line 58. 80–85% of FC was the best fertigation strategy? What do means?

Response 9:Thank you very much for your question.We have supplemented explanations to clarify this statement:

First, define "fertigation" as "an intelligent system integrating water and nutrient delivery" (consistent with Wang et al. (2024) [13]);

Second, specify that "80–85% of FC" refers to the soil moisture control range of this IFS, which was verified as optimal for tomato production in Shouguang, Shandong;

Third, we link this to the research gap: "However, such strategies’ applicability to regions with distinct conditions (e.g., Shijiazhuang, Hebei) is underexplored"—highlighting the necessity of our study.

**Point.10:** Line 75. However, optimizing the application of nitrogen under certain water conditions did not continue to increase yield. Check references [27,28,29,30] and write in a better way.

Response 10:Thank you very much for your question.We have revised this statement to be more precise and evidence-based by citing specific references:

Revised content: "However, Fan et al. (2022) and Zhou et al. (2023) [27,30] showed that under fixed water conditions, N optimization beyond a threshold fails to boost yield—even leading to potential yield decline due to nutrient imbalance; similar findings were reported by Piri et al. (2022) and Zhang et al. (2021) [28,29] in cucumber and grain crops, respectively."

This revision avoids vagueness by: (1) naming key researchers and their findings; (2) explaining the mechanism ("nutrient imbalance"); (3) extending to cross-crop consistency, making the argument more rigorous and aligned with the cited references.

**Point.11:** Fertilization strategies through mathematical statistics methods? Which mathematical statistics methods?

Response 11:Thank you very much for your question.We have replaced the vague "mathematical statistics methods" with specific, manuscript-consistent methods:

Explicitly stated: "highlighting the need for integrated evaluation via principal component analysis (PCA) and entropy-weighted TOPSIS [31-33]";

These methods are not only supported by the cited references [31-33] but also consistent with the "Data Analysis" section of our manuscript (we use PCA for photosynthetic/quality evaluation and entropy-weighted TOPSIS for comprehensive ranking).

This revision ensures methodological clarity and links the introduction to the study’s actual analytical framework.

**Point.12:**The specific objectives were:1. and do not repeat treatments and precise the objectives.

Response 12:Thank you very much for your question.We have revised the 3 objectives to eliminate redundant treatment details (e.g., "75–90% FC vs. 85–90% FC", "0%, 20%, and 40% N reduction") and focus on research logic and outcomes:

Original Objective 1 (repeating treatments): "To evaluate the impacts of two irrigation (75–90% FC vs. 85–90% FC) and two organic fertilizer levels (high vs. low) on tomato parameters, yield, and quality";

Revised Objective 1 (precision): "Evaluate interactions between irrigation lower limits and basal organic fertilizer levels on tomato photosynthetic traits, yield, and quality";

Similarly, we revised Objectives 2 and 3 to emphasize "coupling effects" and "multi-criteria decision analysis (PCA and entropy-weighted TOPSIS)" rather than repeating specific treatment levels.

This revision makes objectives more focused on scientific questions rather than technical details, aligning with academic writing norms.

**Point.13:**”Test Location” change to “Experiment site and detail of greenhouse” OR simply write “Detail of greenhouse”.

Response 13:Thank you very much for your question.We have changed the section title from "Test Location" to "Experiment site and detail of greenhouse".

**Point.14:**Merge headings Test Materials with Experimental Design

Response 14:Thank you very much for your suggestion. We have merged the headings "Test Materials" and "Experimental Design" into "Test Materials with Experimental Design" in accordance with your advice.

**Point.15:** Write treatments in a better way. Treatment mention in T1, T2…….and then L1, L2, N1…etc check out how write treatments in a sound way. Writing efficiency is very poor.In Table 3. Write detail of treatment with Note(S): Different small letters in the same column indicate significant differences among treatments (P< 0.05 level. T1 is N 352.14+ L1:75+ O1:400. Again this will be complicated that what L or O. You have written treatments in a complicated manner.

Response 15: Thank you very much for your suggestion. We have reorganized the presentation of treatments in a clearer manner, with annotations defining each treatment combination (e.g., T1 = O1L1N1). And the details in the notes of Table 3 have been supplemented.

**Point.16:Results**

Here, reading makes complications and will require the reader to go back and see treatments. Otherwise, it will be difficult to determine what does means of T1, T2 etc. The transpiration rate was the highest in T1, with no significant difference among treatments; the net photo-177 synthetic rate was the highest in T8, and there was no significant difference among treatments. The intercellular CO₂ 178 concentration of T9 was the highest, reaching 317.18 µmol・m⁻²・s⁻¹, while T3 was the lowest at 291.98 µmol・m⁻²・179 s⁻¹. Among them, there are significant differences between T9, T1, T3 and T8, while there are no significant differences 180 between other treatments. The stomatal conductance of T12 was the highest, and T3 was the lowest. Except for T3 and 181 T6, there was no obvious difference among other treatments

Response 16: Thank you very much for your suggestion. We have revised the text and annotated all treatment mentions with parenthetical identifiers to enhance reader comprehension of each treatment combination.

**Point.17:** Fig 4. Accumulation of nitrogen, phosphorus and potassium in leaves. Abc ranking is statistically wrong. Also write note treatments what does treatments with T1, T2 all abbreviations represent? There is a problem in Fig 5. Accumulation of nitrogen, phosphorus and potassium in stems. Fig 6. Accumulation of nitrogen, phosphorus and potassium in fruits. Fig 7. Comprehensive ranking of different treatments. This figure nothing tells. Technically wrong. What do colors show?

Response 17: Thank you very much for your suggestion. The format of the figures has been adjusted. The original data will be uploaded to the system as an attachment.The colors in the figure7 represent the relative area occupied by each treatment. The areas are arranged in descending order according to the treatment ranking, with corresponding values displayed. An explanatory note has been added to the document.

**Point.18:** Discussion section and Conclusion
Discuss key results. Check these [39,Error! Reference 370 source not found.].
ONL? What is it? Avoid to many abbreviations in the manuscript.
Response 18: Thank you very much for your suggestion. The citation format of references has been revised. And the abbreviations have been revised.

**Point.19:**In the introduction section, see this manuscript ‘’What should we do for water security? A technical review on more yield per water drop’’ for the paragraph ‘’ Optimal irrigation is critical for maximizing tomato yield and quality while conserving water resources……….Also, see this paper and utilize ‘’Navigating water and nitrogen practices for sustainable wheat production by model-based optimization management systems: A case study of China and Pakistan’’

Response 19: Thank you very much for your suggestion. We have added the references of the two manuscripts, "What should we do for water security? A technical review on more yield per water drop" and "Navigating water and nitrogen practices for sustainable wheat production by model-based optimization management systems: A case study of China and Pakistan", in the introduction section as you advised.

Response to Reviewer 2 comments

**Point.1:** Materials and Methods: Many essential details are missing, and in its current form, the reader cannot replicate the experiments. In fact, it is even difficult to determine whether the methodology adequately addresses the study objectives. The description of the greenhouse is vague and lacks key details about environmental conditions (e.g., temperature control, ventilation, and light transmittance), which are crucial for reproducibility.

Response 1: Thank you very much for your suggestion. We have provided additional details on the core parameters of the experimental greenhouse, including temperature control, ventilation system, and light transmittance.

**Point.2:** High clay content in the soil affects water movement and nutrient availability, but this is not mentioned in the basic properties of soil.

Response 2: Thank you very much for your suggestion. Based on the soil test report and according to the USDA soil texture classification system, the soil in the experimental greenhouse has been identified as loam. The relevant information has been updated in the table.

**Point.3:** In line 98, the author states that “the growth period was 102 days”， is there any basis for this statement? What signifies the end of the experiment?

Response 3: Thank you very much for your suggestion. The 102-day growth cycle from transplanting to final harvest for tomatoes.

**Point.4:** In line 156 , the author mentions the definition of WUE, but uses WUEy in the formula. Does it have the same meaning? I hope the author will correct this.

Response 4: Thank you very much for your suggestion. Both have the same meaning, and "y" has been removed.

**Point.5:** The Results section reiterates data already presented in the tables, failing to highlight key findings. I recommend that the authors streamline the description and prioritize significant differences and interaction effects.

Response 5: Thank you very much for your suggestion.The conclusions have been streamlined to highlight significant differences and interaction effects.For details, please see the original text.

**Point.6:** The current discussion focuses primarily on describing the results, lacking in-depth comparisons with existing studies and mechanistic explanations. The authors are advised to provide more explanations for any discrepancies between the results and the literature (e.g., the possible reasons for the non-significant interaction between ON and LN) and to explore the mechanisms of water-fertilizer coupling in the soil-plant system.

Response 6: Thank you very much for your suggestion.The Discussion section has been revised as suggested.For details, please see the original text.

**Point.7:** The language needs further polishing. The “lower limit of irrigation”should be unified as “irrigation threshold” or “lower limit of irrigation”. It is recommended that a native English speaker polish the entire text to ensure accurate terminology and fluent sentences.

Response 7: Thank you very much for your suggestion. The terms have been standardized as “lower limit of irrigatio”

**Point.8:** The reference format is not uniform, please correct it.

Response 8: Thank you very much for your suggestion. The format of references has been revised.
