# Supplementary material for: Effects of different irrigation lower limits and combined application of reduced nitrogen with organic fertilizer on spring-season tomatoes (Solanum lycopersicum)
Source: PLoS One. 2026 Aug 11;21(8):e0354927. doi: 10.1371/journal.pone.0354927 (PMC13460604; doi:10.1371/journal.pone.0354927)
Supplement: Supplementary file 4 [file pone.0354927.s004.docx]

**Reviewer comments**

1. Some references in the manuscript display “[Error! Reference source not found.,]”, the author needs to verify and revise.

2. The abstract states three-way ANOVA, but the methods section states one-way ANOVA. The statistical model used needs to be consistent and clearly defined.

3. The experimental design lacks reproducibility. Key information such as irrigation triggering method, single and cumulative irrigation volume, number of topdressing applications, and specific timing is still insufficiently detailed.

4. Experimental replication is unclear. The text does not clearly state how many independent replicates each treatment has, or whether plots are statistical units.

5.The description of the comprehensive evaluation method is too simplistic. PCA and entropy-weighted TOPSIS are key bases for deriving the “optimal treatment” but the related calculation procedures are not clearly explained.

6.Figures and tables still need optimization. The text “Fig 7. Comprehensive ranking of different treatments.” is not very readable; it is recommended to present the ranking and scores in a more intuitive way.

7.It is recommended that a native English speaker polish the entire text to ensure accurate terminology and fluent sentences.
