# Supplementary material for: Effects of different irrigation lower limits and combined application of reduced nitrogen with organic fertilizer on spring-season tomatoes (Solanum lycopersicum)
Source: PLoS One. 2026 Aug 11;21(8):e0354927. doi: 10.1371/journal.pone.0354927 (PMC13460604; doi:10.1371/journal.pone.0354927)
Supplement: Supplementary file 5 [file pone.0354927.s005.docx]

**Response to Reviewer 1comments**

**Point.1:** Some references in the manuscript display “[Error! Reference source not found.,]”, the author needs to verify and revise.

**Response 1:** Thank you very much for your suggestion. The citation format of references has been revised.

**Point.2:** The abstract states three-way ANOVA, but the methods section states one-way ANOVA. The statistical model used needs to be consistent and clearly defined.

**Response 2:** Thank you very much for your suggestion. The statistical models in the paper have been kept consistent.

**Point.3:** The experimental design lacks reproducibility. Key information such as irrigation triggering method, single and cumulative irrigation volume, number of topdressing applications, and specific timing is still insufficiently detailed.

**Response 3:** Thank you very much for your suggestion. The key experimental information has been supplemented and explained in the sections "Test Materials and Experimental Design" and "Environmental Conditions and Water Consumption in Solar Greenhouse".

**Point.4:** Experimental replication is unclear. The text does not clearly state how many independent replicates each treatment has, or whether plots are statistical units.

**Response 4:** Thank you very much for your suggestion. We apologize for the unclear description of experimental replication. Each treatment included three independent biological replicates, and each plot was regarded as the basic statistical unit for ANOVA and multiple comparison analysis. Relevant details have been clearly supplemented in the Materials and Methods section of the revised manuscript.

**Point.5:** The description of the comprehensive evaluation method is too simplistic. PCA and entropy-weighted TOPSIS are key bases for deriving the “optimal treatment” but the related calculation procedures are not clearly explained.

**Response 5:** Thank you very much for your suggestion. We sincerely appreciate the constructive suggestion. To clarify the comprehensive evaluation procedures, we have supplemented the detailed calculation formulas and implementation steps of Principal Component Analysis (PCA) and Entropy-weighted TOPSIS in the Data Analysis section of the revised manuscript. The complete calculation process including data standardization, eigenvalue solving, weight determination, distance calculation and comprehensive scoring has been clearly presented, which fully supports the reliability of the optimal treatment screening result.

**Point.6:** Figures and tables still need optimization. The text “Fig 7. Comprehensive ranking of different treatments.” is not very readable; it is recommended to present the ranking and scores in a more intuitive way.

**Response 6:** Thank you very much for your suggestion. The figure is replaced by a more intuitive table.

**Point.7:** It is recommended that a native English speaker polish the entire text to ensure accurate terminology and fluent sentences.

**Response 7:** Thank you very much for your suggestion. The entire manuscript has been carefully polished and revised by a professional native English editing service to ensure accurate scientific terminology, proper grammar, and fluent expression. We believe the language quality of the revised manuscript has been significantly improved.

**Response to Reviewer 2comments**

**Point.1:** Please perform a minor revision. Does L2 increase tomato yield? Please also write increase yield in the abstract. Also, clarify the chemical nitrogen fertilization application in L2.

**Response 1:** Thank you very much for your suggestion.L2 can increase tomato yield, and relevant explanations of yield increase have been supplemented in the abstract. The application of chemical nitrogen fertilizer under each L2 treatment can be found in Table 2 in the text.

**Point.2:** Please check out citations problem in the whole manuscript ‘’Error! Reference source not found.,Error! Reference source’’

**Response 2:** Thank you very much for your suggestion. The citation format of references has been revised.

**Point.3:** Correct caption of Fig 1.Tomato growth stages.Including growth images of four periods：transplanting、seeding、florescence、harvest

**Response 3:** Thank you very much for your suggestion. Both the images and the caption in Figure 1 have been revised.

**Point.4:** Yield(kg/Plant) in the table, please could you add the yield in kg/ha ?

**Response 4:**We appreciate your valuable suggestion. However, we do not plan to modify the table to add the yield in kg/ha, as the current unit (kg/Plant) is more consistent with the research focus and data analysis logic of this study.

**Point.5:** Note(s): “*” indicates a significant correlation at the p < 0.05 level, and “**” indicates a highly significant correlationat the p < 0.01 level. In Table, write full forms of Index. For example, with note, write: Note(s): “*” indicates a significant correlation at the p < 0.05 level, and “**” indicates a highly significant correlationat the p < 0.01 level. U1 is xyz, U2 is xyz, ………..F6 is xyz.

**Response 5:** Thank you very much for your suggestion. The notes for Table 7 have been revised and completed.

**Point.6:** Table 10. Scores and rankings of Tier 1 indicators. Write note For T1, T2, T3,In conclusion, please clearly state which treatment combination is the optimum choice for the highest yield and quality of Tomato.

**Response 6:** Thank you very much for your suggestion. The notes for T1, T2, and T3 in Table 10 have been added. Information on the optimal treatment combination has been supplemented in the conclusion.
