# Supplementary material for: Effects of different irrigation lower limits and combined application of reduced nitrogen with organic fertilizer on spring-season tomatoes (Solanum lycopersicum)
Source: PLoS One. 2026 Aug 11;21(8):e0354927. doi: 10.1371/journal.pone.0354927 (PMC13460604; doi:10.1371/journal.pone.0354927)
Supplement: Supplementary file 6 [file pone.0354927.s006.docx]

**Reviewer Comments**

The manuscript has been revised and improved in response to the expert comments, and the authors have addressed the reviewers' questions appropriately. However, the following issues remain:

1. The authors measured soil field capacity, but the specific values are not provided in the text. Please supplement this information.
2. In Table 1, the soil type is given as loam, but the specific particle size distribution is not provided. Please provide this information.
3. The basis for the organic fertilizer application rates is missing. Please provide the rationale for the chosen rates.
4. In Equation (1), "total" should be written as a subscript.
5. Although transpiration rate and net photosynthetic rate showed no significant differences among treatments, intercellular CO₂ concentration and stomatal conductance did show significant differences. Please examine whether this has been explained in the Discussion section.
6. Lines 374–377 state: *"However, except for a few treatments, there were no obvious differences in photosynthetic indices between different treatments, which may be attributed to the imprecise timing of photosynthetic measurements, leading to convergent leaf photosynthetic capacity under multi-factor coupling."* Attributing the lack of differences to measurement timing seems somewhat tenuous. Is there any literature to support this statement?
7. In the Conclusions, the phrase *"setting the soil irrigation level at 85% FC to 90% FC"* is not accurate. Should it instead read as setting the **upper limit** of irrigation at 85–90% FC?
