# Supplementary material for: Effects of different irrigation lower limits and combined application of reduced nitrogen with organic fertilizer on spring-season tomatoes (Solanum lycopersicum)
Source: PLoS One. 2026 Aug 11;21(8):e0354927. doi: 10.1371/journal.pone.0354927 (PMC13460604; doi:10.1371/journal.pone.0354927)
Supplement: Peer review history

# Peer review: Decision Letter 0

**DOI:** 10.1371/journal.pone.0354927.r001

**Authors:** Umesh Sharma

11 Oct 2025

Dear Dr. li,

Thank you for submitting your manuscript to PLOS ONE. After careful consideration, we feel that it has merit but does not fully meet PLOS ONE’s publication criteria as it currently stands. Therefore, we invite you to submit a revised version of the manuscript that addresses the points raised during the review process.

Please submit your revised manuscript by Nov 25 2025 11:59PM. If you will need more time than this to complete your revisions, please reply to this message or contact the journal office at plosone@plos.org. When you're ready to submit your revision, log on to https://www.editorialmanager.com/pone/ and select the 'Submissions Needing Revision' folder to locate your manuscript file.

- A rebuttal letter that responds to each point raised by the academic editor and reviewer(s). You should upload this letter as a separate file labeled 'Response to Reviewers'.
- A marked-up copy of your manuscript that highlights changes made to the original version. You should upload this as a separate file labeled 'Revised Manuscript with Track Changes'.
- An unmarked version of your revised paper without tracked changes. You should upload this as a separate file labeled 'Manuscript'.

If you would like to make changes to your financial disclosure, please include your updated statement in your cover letter. Guidelines for resubmitting your figure files are available below the reviewer comments at the end of this letter.

If applicable, we recommend that you deposit your laboratory protocols in protocols.io to enhance the reproducibility of your results. Protocols.io assigns your protocol its own identifier (DOI) so that it can be cited independently in the future. For instructions see: https://journals.plos.org/plosone/s/submission-guidelines#loc-laboratory-protocols. Additionally, PLOS ONE offers an option for publishing peer-reviewed Lab Protocol articles, which describe protocols hosted on protocols.io. Read more information on sharing protocols at https://plos.org/protocols?utm_medium=editorial-email&utm_source=authorletters&utm_campaign=protocols.

We look forward to receiving your revised manuscript.

Kind regards,

Umesh Sharma

Academic Editor

PLOS ONE

Journal Requirements:

When submitting your revision, we need you to address these additional requirements.

1. Please ensure that your manuscript meets PLOS ONE's style requirements, including those for file naming. The PLOS ONE style templates can be found at https://journals.plos.org/plosone/s/file?id=wjVg/PLOSOne_formatting_sample_main_body.pdf and https://journals.plos.org/plosone/s/file?id=ba62/PLOSOne_formatting_sample_title_authors_affiliations.pdf

2. In your Methods section, please provide additional information regarding the permits you obtained for the work. Please ensure you have included the full name of the authority that approved the field site access and, if no permits were required, a brief statement explaining why.

3. We note that the grant information you provided in the ‘Funding Information’ and ‘Financial Disclosure’ sections do not match.

When you resubmit, please ensure that you provide the correct grant numbers for the awards you received for your study in the ‘Funding Information’ section.

4. Thank you for stating the following financial disclosure:

Hebei Provincial Fund Project for the Transformation of Agricultural Scientific and Technological Achievements(2025JNZ-A11);Shijiazhuang Science and Technology Program Project (231490361).

Please state what role the funders took in the study. If the funders had no role, please state: "The funders had no role in study design, data collection and analysis, decision to publish, or preparation of the manuscript."

If this statement is not correct you must amend it as needed.

Please include this amended Role of Funder statement in your cover letter; we will change the online submission form on your behalf.

5. We note that Figure 1 in your submission contain copyrighted images. All PLOS content is published under the Creative Commons Attribution License (CC BY 4.0), which means that the manuscript, images, and Supporting Information files will be freely available online, and any third party is permitted to access, download, copy, distribute, and use these materials in any way, even commercially, with proper attribution. For more information, see our copyright guidelines: http://journals.plos.org/plosone/s/licenses-and-copyright.

We require you to either (1) present written permission from the copyright holder to publish these figures specifically under the CC BY 4.0 license, or (2) remove the figures from your submission:

a. You may seek permission from the original copyright holder of Figure 1 to publish the content specifically under the CC BY 4.0 license.

We recommend that you contact the original copyright holder with the Content Permission Form (http://journals.plos.org/plosone/s/file?id=7c09/content-permission-form.pdf) and the following text:

“I request permission for the open-access journal PLOS ONE to publish XXX under the Creative Commons Attribution License (CCAL) CC BY 4.0 (http://creativecommons.org/licenses/by/4.0/). Please be aware that this license allows unrestricted use and distribution, even commercially, by third parties. Please reply and provide explicit written permission to publish XXX under a CC BY license and complete the attached form.”

Please upload the completed Content Permission Form or other proof of granted permissions as an "Other" file with your submission.

In the figure caption of the copyrighted figure, please include the following text: “Reprinted from [ref] under a CC BY license, with permission from [name of publisher], original copyright [original copyright year].”

b. If you are unable to obtain permission from the original copyright holder to publish these figures under the CC BY 4.0 license or if the copyright holder’s requirements are incompatible with the CC BY 4.0 license, please either i) remove the figure or ii) supply a replacement figure that complies with the CC BY 4.0 license. Please check copyright information on all replacement figures and update the figure caption with source information. If applicable, please specify in the figure caption text when a figure is similar but not identical to the original image and is therefore for illustrative purposes only.

6. Please include captions for your Supporting Information files at the end of your manuscript, and update any in-text citations to match accordingly. Please see our Supporting Information guidelines for more information: http://journals.plos.org/plosone/s/supporting-information.

7. If the reviewer comments include a recommendation to cite specific previously published works, please review and evaluate these publications to determine whether they are relevant and should be cited. There is no requirement to cite these works unless the editor has indicated otherwise.

[Note: HTML markup is below. Please do not edit.]

Reviewers' comments:

Reviewer's Responses to Questions

**Comments to the Author**

1. Is the manuscript technically sound, and do the data support the conclusions?

Reviewer #1: Yes

Reviewer #2: Yes

**********

2. Has the statistical analysis been performed appropriately and rigorously?

Reviewer #1: Yes

Reviewer #2: Yes

**********

3. Have the authors made all data underlying the findings in their manuscript fully available?

The PLOS Data policy

Reviewer #1: Yes

Reviewer #2: Yes

**********

4. Is the manuscript presented in an intelligible fashion and written in standard English?

Reviewer #1: Yes

Reviewer #2: No

**********

Reviewer #1: This study systematically explored the effects of three factors (irrigation minimum, organic fertilizer application rate, and nitrogen fertilizer reduction) on the photosynthetic characteristics, yield, quality, and water and nitrogen use efficiency of greenhouse spring tomatoes through a crossover experiment. Principal component analysis and the entropy-weighted TOPSIS method were used for comprehensive evaluation. The study design was sound, the analytical methods were appropriate, and it offers practical guidance, particularly in the area of water-fertilizer coordination. However, the manuscript requires further improvement in terms of experimental design, interpretation of results, and language .

Reviewer #2: The manuscript is interesting and suitable for the journal. However, a major revision is required. Authors should show high writing efficiency. Following suggestions and comments would be considered.

Title and abstract section.

The effects of different irrigation lower limits and combined application of reduced nitrogen with organic fertilizer on spring-season tomato.

Improve the title OR just remove ‘’The’’. Effects of different irrigation lower limits and combined application of reduced nitrogen with organic fertilizer on spring-season tomato.

haijie li check the author name. First characters should be capitalized

In abstract, write one to two sentences of context or the importance of the study.

This is too long. To investigate the effects of different lower irrigation limits, reduced nitrogen application combined with organic 23 fertilizer on photosynthetic parameters, yield, quality, and water-nitrogen use efficiency of spring greenhouse tomatoes, 24 and to provide a scientific water and fertilizer management system for local tomato cultivation, spring greenhouse 25 tomatoes were used as test materials.

What was the irrigation amount (mm) in each treatment? Write irrigation amount for irrigation levels 75%FC�90%FC (L1) and 85%FC�90%FC (L2).

When was the experiment conducted? Is it one year or two year or more? English writing should be improved in the abstract; it needs to be rewritten. For topdressing, nitrate nitrogen was 28 applied with three nitrogen levels: conventional nitrogen rate (N1, 352.14 kg·hm⁻²), 20% reduced nitrogen (N2, 281.72 29 kg·hm⁻²), and 40% reduced nitrogen (N3, 211.29 kg·hm⁻²). A three-factor cross experiment was conducted.

The whole abstract is composed of the materials and methods section. The context is missing and the results are not detailed. The results showed that applying a lower level of organic 33 fertilizer, reducing nitrogen application by 20%, and setting the irrigation level at 85%FC�90%FC could maximize the comprehensive benefits of tomato growth.

Introduction section

The research gap should be better emphasized. Avoid unnecessary wording and sentences. Completed the introduction section in three paragraphs. The longer the introduction section higher the mistakes and the lower the writing efficiency.

Avoid repetition of study area ‘’Shandong, Hebei, and Henan’’, Shijiazhuang, Hebei.

Line 58. 80–85% of FC was the best fertigation strategy? What do means?

Line 75. However, optimizing the application of nitrogen under certain water conditions did not continue to increase yield. Check references [27,28,29,30] and write in a better way.

fertilization strategies through mathematical statistics methods? Which mathematical statistics methods?

The specific objectives were:1. and do not repeat treatments and precise the objectives.

Materials and Methods

Test Location change to ‘’Experiment site and detail of greenhouse’’ OR simply write ‘’ Detail of greenhouse’’. The experiment was conducted………..

Merge headings Test Materials with Experimental Design

Write treatments in a better way. Treatment mention in T1, T2…….and then L1, L2, N1…etc check out how write treatments in a sound way. Writing efficiency is very poor.

In Table 3. Write detail of treatment with Note(S): Different small letters in the same column indicate significant differences among treatments (P< 0.05 level. T1 is N 352.14+ L1:75+ O1:400. Again this will be complicated that what L or O. You have written treatments in a complicated manner.

Check equations of WUE and NUE

Results

Here, reading makes complications and will require the reader to go back and see treatments. Otherwise, it will be difficult to determine what does means of T1, T2 etc. The transpiration rate was the highest in T1, with no significant difference among treatments; the net photo-177 synthetic rate was the highest in T8, and there was no significant difference among treatments. The intercellular CO₂ 178 concentration of T9 was the highest, reaching 317.18 µmol・m⁻²・s⁻¹, while T3 was the lowest at 291.98 µmol・m⁻²・179 s⁻¹. Among them, there are significant differences between T9, T1, T3 and T8, while there are no significant differences 180 between other treatments. The stomatal conductance of T12 was the highest, and T3 was the lowest. Except for T3 and 181 T6, there was no obvious difference among other treatments

Fig 4. Accumulation of nitrogen, phosphorus and potassium in leaves. Abc ranking is statistically wrong. Also write note treatments what does treatments with T1, T2 all abbreviations represent? There is a problem in Fig 5. Accumulation of nitrogen, phosphorus and potassium in stems. Fig 6. Accumulation of nitrogen, phosphorus and potassium in fruits.

Fig 7. Comprehensive ranking of different treatments. This figure nothing tells. Technically wrong. What do colors show?

Discussion section and Conclusion

Discuss key results. Check these [39,Error! Reference 370 source not found.].

ONL? What is it? Avoid to many abbreviations in the manuscript.

In the introduction section, see this manuscript ‘’What should we do for water security? A technical review on more yield per water drop’’ for the paragraph ‘’ Optimal irrigation is critical for maximizing tomato yield and quality while conserving water resources……….Also, see this paper and utilize ‘’Navigating water and nitrogen practices for sustainable wheat production by model-based optimization management systems: A case study of China and Pakistan’’

**********

what does this mean?). If published, this will include your full peer review and any attached files.

If you choose “no”, your identity will remain anonymous but your review may still be made public.

**Do you want your identity to be public for this peer review?**  For information about this choice, including consent withdrawal, please see our Privacy Policy

Reviewer #1: No

Reviewer #2: No

**********

[NOTE: If reviewer comments were submitted as an attachment file, they will be attached to this email and accessible via the submission site. Please log into your account, locate the manuscript record, and check for the action link "View Attachments". If this link does not appear, there are no attachment files.]

While revising your submission, please upload your figure files to the Preflight Analysis and Conversion Engine (PACE) digital diagnostic tool, https://pacev2.apexcovantage.com/. PACE helps ensure that figures meet PLOS requirements. To use PACE, you must first register as a user. Registration is free. Then, login and navigate to the UPLOAD tab, where you will find detailed instructions on how to use the tool. If you encounter any issues or have any questions when using PACE, please email PLOS at figures@plos.org. Please note that Supporting Information files do not need this step.

Attachment Submitted filename: PONE-D-25-44070-Reviewer comments.docx

# Author comment: Author response to Decision Letter 1

**DOI:** 10.1371/journal.pone.0354927.r002

3 Mar 2026

Response to Reviewer 1comments

Point.1: The effects of different irrigation lower limits and combined application ofreduced nitrogen with organic fertilizer on spring-season tomato.Improve the title OR just remove ‘’The’’. Effects of different irrigation lower limits and combined application of reduced nitrogen with organic fertilizer on spring-season tomato.

Response 1: Thank you very much for your suggestion. The revised title "Greenhouse tomato is a high-value cash crop globally, but inefficient water and nitrogen management in greenhouse systems often causes resource waste, environmental risks, and reduced yield/quality—key challenges for sustainable cultivation." is more concise and in line with the academic expression norms of journal titles, and we have updated it in the manuscript accordingly.

Point.2: haijie li check the author name. First characters should be capitalized.

Response 2: Thank you very much for your question. We have revised the author name to "Haijie Li" in accordance with your suggestion that the first characters of both the given name and surname should be capitalized.

Point.3: In abstract, write one to two sentences of context or the importance of the study.

This is too long.

Response 3:Thank you very much for your question.We have supplemented one background sentence at the beginning of the abstract to clarify the research significance: “Greenhouse tomato is a key cash crop in many regions, but irrational water and fertilizer management often leads to low resource use efficiency and compromised yield/quality.” This sentence highlights the practical problem addressed by the study, making the research purpose more targeted.

Point.4: What was the irrigation amount (mm) in each treatment? Write irrigation amount for irrigation levels 75%FC�90%FC (L1)and 85%FC�90%FC (L2).

Response 4:Thank you very much for your question.We set the irrigation amounts based on soil moisture content measured by soil moisture sensors, with reference to soil field capacity. Due to the spatial heterogeneity of soil, the field capacity varies across different locations, resulting in differences in the actual irrigation amounts among treatments even under the same field capacity-based settings. The specific irrigation amounts for each treatment are detailed in Table 5. Therefore, we have revised the text by adding "where FC refers to soil field capacity" after the sentence "Two irrigation levels were set: 75%FC�90%FC (L1) and 85%FC�90%FC (L2)" to facilitate readers' understanding.

Point.5: When was the experiment conducted? Is it one year or two year or more?

Response 5:Thank you for your question regarding the experimental timeline. We have incorporated the relevant information into the abstract to clarify the duration and timing of the experiment.

The experiment was conducted in 2024, with the tomatoes planted on March 14, the formal experiment initiation on April 1, a total growth period of 102 days, and vine pulling completed on June 24. This specifies that the study was based on a one-year field trial, as detailed in the revised abstract.

Point.6: English writing should be improved in the abstract; it needs to be rewritten.For topdressing, nitrate nitrogen was applied with three nitrogen levels: conventional nitrogen rate (N1, 352.14 kg·hm⁻²), 20% reduced nitrogen (N2, 281.72 kg·hm⁻²), and 40% reduced nitrogen (N3, 211.29 kg·hm⁻²). A three-factor cross experiment was conducted.

The whole abstract is composed of the materials and methods section. The context is missing and the results are not detailed. The results showed that applying a lower level of organic fertilizer, reducing nitrogen application by 20%, and setting the irrigation level at 85%FC�90%FC could maximize the comprehensive benefits of tomato growth.

Response 6:Thank you for your detailed comments on the abstract. We fully agree with your suggestions to improve the English expression, balance the structure, and enrich the results. We have revised the abstract accordingly:

Enhanced English writing: We refined awkward phrases (e.g., revised "For topdressing, nitrate nitrogen was applied with three nitrogen levels" to "three topdressing nitrate nitrogen levels") .

Strengthened context: We added background on sustainable cultivation challenges (resource waste, environmental risks) to clarify the study’s significance, addressing the "missing context" concern.

Detailed results: We replaced the vague description of "comprehensive benefits" with specific findings of treatment T11, including its highest values for vitamin C (21.46 mg/100g) and soluble solids content (5.62%), as well as the highest total nitrogen content in fruits, stems, and leaves (62.83% higher than the lowest treatment).

We clarified the application of principal component analysis (for photosynthetic and quality indicators) and the entropy-weighted TOPSIS method, explicitly listing the first-level evaluation indicators (F1–F6) and confirming that T11 ranked first in the comprehensive ranking

Point.7: The research gap should be better emphasized. Avoid unnecessary wording and sentences. Completed the introduction section in threeparagraphs. The longer the introduction section higher the mistakes and the lower the writing efficiency.

Response 7:Thank you very much for your question.We have restructured the introduction into 3 concise paragraphs, removed redundant content (e.g., redundant descriptions of greenhouse advantages, repetitive mentions of fertilizer types), and explicitly highlighted 3 core research gaps:

Regional applicability gap: Existing irrigation strategies (e.g., 70% FC in Huaibei, 80–85% FC in Shouguang) lack verification in Shijiazhuang with distinct soil-climatic conditions;

Interaction gap: Optimal ratio of organic-to-inorganic fertilizers and its coupling with irrigation remain unclear;

Evaluation method gap: Single indices are insufficient, requiring integrated methods (PCA, entropy-weighted TOPSIS) for comprehensive assessment.

This revision improves writing efficiency and reduces potential errors by streamlining logic and removing non-essential details.

Point.8: Avoid repetition of study area ‘’Shandong, Hebei, and Henan’’, Shijiazhuang, Hebei.

Response 8:Thank you very much for your question.We have optimized the mention of study areas to avoid repetition:

In Paragraph 1, we only note "Shandong, Hebei, and Henan as core planting provinces" to clarify China’s tomato production layout, without redundant elaboration;

In Paragraph 2, we contrast existing research regions (Huaibei, Shouguang) with the target region (Shijiazhuang) only once, focusing on the "regional applicability gap" rather than repeating "Hebei" separately;

No further redundant mentions of these regions appear in Paragraph 3.

This ensures clarity of regional context while eliminating repetitive content.

Point.9: Line 58. 80–85% of FC was the best fertigation strategy? What do means?

Response 9:Thank you very much for your question.We have supplemented explanations to clarify this statement:

First, define "fertigation" as "an intelligent system integrating water and nutrient delivery" (consistent with Wang et al. (2024) [13]);

Second, specify that "80–85% of FC" refers to the soil moisture control range of this IFS, which was verified as optimal for tomato production in Shouguang, Shandong;

Third, we link this to the research gap: "However, such strategies’ applicability to regions with distinct conditions (e.g., Shijiazhuang, Hebei) is underexplored"—highlighting the necessity of our study.

Point.10: Line 75. However, optimizing the application of nitrogen under certain water conditions did not continue to increase yield. Check references [27,28,29,30] and write in a better way.

Response 10:Thank you very much for your question.We have revised this statement to be more precise and evidence-based by citing specific references:

Revised content: "However, Fan et al. (2022) and Zhou et al. (2023) [27,30] showed that under fixed water conditions, N optimization beyond a threshold fails to boost yield—even leading to potential yield decline due to nutrient imbalance; similar findings were reported by Piri et al. (2022) and Zhang et al. (2021) [28,29] in cucumber and grain crops, respectively."

This revision avoids vagueness by: (1) naming key researchers and their findings; (2) explaining the mechanism ("nutrient imbalance"); (3) extending to cross-crop consistency, making the argument more rigorous and aligned with the cited references.

Point.11: Fertilization strategies through mathematical statistics methods? Which mathematical statistics methods?

Response 11:Thank you very much for your question.We have replaced the vague "mathematical statistics methods" with specific, manuscript-consistent methods:

Explicitly stated: "highlighting the need for integrated evaluation via principal component analysis (PCA) and entropy-weighted TOPSIS [31-33]";

These methods are not only supported by the cited references [31-33] but also consistent with the "Data Analysis" section of our manuscript (we use PCA for photosynthetic/quality evaluation and entropy-weighted TOPSIS for comprehensive ranking).

This revision ensures methodological clarity and links the introduction to the study’s actual analytical framework.

Point.12:The specific objectives were:1. and do not repeat treatments and precise the objectives.

Response 12:Thank you very much for your question.We have revised the 3 objectives to eliminate redundant treatment details (e.g., "75–90% FC vs. 85–90% FC", "0%, 20%, and 40% N reduction") and focus on research logic and outcomes:

Original Objective 1 (repeating treatments): "To evaluate the impacts of two irrigation (75–90% FC vs. 85–90% FC) and two organic fertilizer levels (high vs. low) on tomato parameters, yield, and quality";

Revised Objective 1 (precision): "Evaluate interactions between irrigation lower limits and basal organic fertilizer levels on tomato photosynthetic traits, yield, and quality";

Similarly, we revised Objectives 2 and 3 to emphasize "coupling effects" and "multi-criteria decision analysis (PCA and entropy-weighted TOPSIS)" rather than repeating specific treatment levels.

This revision makes objectives more focused on scientific questions rather than technical details, aligning with academic writing norms.

Point.13:”Test Location” change to “Experiment site and detail of greenhouse” OR simply write “Detail of greenhouse”.

Response 13:Thank you very much for your question.We have changed the section title from "Test Location" to "Experiment site and detail of greenhouse".

Point.14:Merge headings Test Materials with Experimental Design

Response 14:Thank you very much for your suggestion. We have merged the headings "Test Materials" and "Experimental Design" into "Test Materials with Experimental Design" in accordance with your advice.

Point.15: Write treatments in a better way. Treatment mention in T1, T2…….and then L1, L2, N1…etc check out how write treatments in a sound way. Writing efficiency is very poor.In Table 3. Write detail of treatment with Note(S): Different small letters in the same column indicate significant differences among treatments (P< 0.05 level. T1 is N 352.14+ L1:75+ O1:400. Again this will be complicated that what L or O. You have written treatments in a complicated manner.

Response 15: Thank you very much for your suggestion. We have reorganized the presentation of treatments in a clearer manner, with annotations defining each treatment combination (e.g., T1 = O1L1N1). And the details in the notes of Table 3 have been supplemented.

Point.16:Results

Here, reading makes complications and will require the reader to go back and see treatments. Otherwise, it will be difficult to determine what does means of T1, T2 etc. The transpiration rate was the highest in T1, with no significant difference among treatments; the net photo-177 synthetic rate was the highest in T8, and there was no significant difference among treatments. The intercellular CO₂ 178 concentration of T9 was the highest, reaching 317.18 µmol・m⁻²・s⁻¹, while T3 was the lowest at 291.98 µmol・m⁻²・179 s⁻¹. Among them, there are significant differences between T9, T1, T3 and T8, while there are no significant differences 180 between other treatments. The stomatal conductance of T12 was the highest, and T3 was the lowest. Except for T3 and 181 T6, there was no obvious difference among other treatments

Response 16: Thank you very much for your suggestion. We have revised the text and annotated all treatment mentions with parenthetical identifiers to enhance reader comprehension of each treatment combination.

Point.17: Fig 4. Accumulation of nitrogen, phosphorus and potassium in leaves. Abc ranking is statistically wrong. Also write note treatments what does treatments with T1, T2 all abbreviations represent? There is a problem in Fig 5. Accumulation of nitrogen, phosphorus and potassium in stems. Fig 6. Accumulation of nitrogen, phosphorus and potassium in fruits. Fig 7. Comprehensive ranking of different treatments. This figure nothing tells. Technically wrong. What do colors show?

Response 17: Thank you very much for your suggestion. The format of the figures has been adjusted. The original data will be uploaded to the system as an attachment.The colors in the figure7 represent the relative area occupied by each treatment. The areas are arranged in descending order according to the treatment ranking, with corresponding values displayed. An explanatory note has been added to the document.

Point.18: Discussion section and Conclusion

Discuss key results. Check these [39,Error! Reference 370 source not found.].

ONL? What is it? Avoid to many abbreviations in the manuscript.

Response 18: Thank you very much for your suggestion. The citation format of references has been revised. And the abbreviations have been revised.

Point.19:In the introduction section, see this manuscript ‘’What should we do for water security? A technical review on more yield per water drop’’ for the paragraph ‘’ Optimal irrigation is critical for maximizing tomato yield and quality while conserving water resources……….Also, see this paper and utilize ‘’Navigating water and nitrogen practices for sustainable wheat production by model-based optimization management systems: A case study of China and Pakistan’’

Response 19: Thank you very much for your suggestion. We have added the references of the two manuscripts, "What should we do for water security? A technical review on more yield per water drop" and "Navigating water and nitrogen practices for sustainable wheat production by model-based optimization management systems: A case study of China and Pakistan", in the introduction section as you advised.

Response to Reviewer 2 comments

Point.1: Materials and Methods: Many essential details are missing, and in its current form, the reader cannot replicate the experiments. In fact, it is even difficult to determine whether the methodology adequately addresses the study objectives. The description of the greenhouse is vague and lacks key details about environmental conditions (e.g., temperature control, ventilation, and light transmittance), which are crucial for reproducibility.

Response 1: Thank you very much for your suggestion. We have provided additional details on the core parameters of the experimental greenhouse, including temperature control, ventilation system, and light transmittance.

Point.2: High clay content in the soil affects water movement and nutrient availability, but this is not mentioned in the basic properties of soil.

Response 2: Thank you very much for your suggestion. Based on the soil test report and according to the USDA soil texture classification system, the soil in the experimental greenhouse has been identified as loam. The relevant information has been updated in the table.

Point.3: In line 98, the author states that “the growth period was 102 days”� is there any basis for this statement? What signifies the end of the experiment?

Response 3: Thank you very much for your suggestion. The 102-day growth cycle from transplanting to final harvest for tomatoes.

Point.4: In line 156 , the author mentions the definition of WUE, but u

Attachment Submitted filename: Response to Reviewers.docx

# Peer review: Decision Letter 1

**DOI:** 10.1371/journal.pone.0354927.r003

**Authors:** Umesh Sharma

23 Mar 2026

Thank you for submitting your manuscript to PLOS ONE. After careful consideration, we feel that it has merit but does not fully meet PLOS ONE’s publication criteria as it currently stands. Therefore, we invite you to submit a revised version of the manuscript that addresses the points raised during the review process.

Please submit your revised manuscript by May 07 2026 11:59PM. If you will need more time than this to complete your revisions, please reply to this message or contact the journal office at plosone@plos.org. When you're ready to submit your revision, log on to https://www.editorialmanager.com/pone/ and select the 'Submissions Needing Revision' folder to locate your manuscript file.

- A letter that responds to each point raised by the academic editor and reviewer(s). You should upload this letter as a separate file labeled 'Response to Reviewers'.
- A marked-up copy of your manuscript that highlights changes made to the original version. You should upload this as a separate file labeled 'Revised Manuscript with Track Changes'.
- An unmarked version of your revised paper without tracked changes. You should upload this as a separate file labeled 'Manuscript'.

If you would like to make changes to your financial disclosure, please include your updated statement in your cover letter. Guidelines for resubmitting your figure files are available below the reviewer comments at the end of this letter.

If applicable, we recommend that you deposit your laboratory protocols in protocols.io to enhance the reproducibility of your results. Protocols.io assigns your protocol its own identifier (DOI) so that it can be cited independently in the future. For instructions see: https://journals.plos.org/plosone/s/submission-guidelines#loc-laboratory-protocols. Additionally, PLOS ONE offers an option for publishing peer-reviewed Lab Protocol articles, which describe protocols hosted on protocols.io. Read more information on sharing protocols at https://plos.org/protocols?utm_medium=editorial-email&utm_source=authorletters&utm_campaign=protocols.

We look forward to receiving your revised manuscript.

Kind regards,

Umesh Sharma

Academic Editor

PLOS One

Journal Requirements:

If the reviewer comments include a recommendation to cite specific previously published works, please review and evaluate these publications to determine whether they are relevant and should be cited. There is no requirement to cite these works unless the editor has indicated otherwise.

Reviewers' comments:

Reviewer's Responses to Questions

**Comments to the Author**

Reviewer #1: (No Response)

Reviewer #2: All comments have been addressed

**********

2. Is the manuscript technically sound, and do the data support the conclusions?

Reviewer #1: Yes

Reviewer #2: Yes

**********

3. Has the statistical analysis been performed appropriately and rigorously?

Reviewer #1: Yes

Reviewer #2: Yes

**********

4. Have the authors made all data underlying the findings in their manuscript fully available?

The PLOS Data policy

Reviewer #1: Yes

Reviewer #2: Yes

**********

5. Is the manuscript presented in an intelligible fashion and written in standard English?

Reviewer #1: No

Reviewer #2: Yes

**********

Reviewer #1: The author has made revisions to the manuscript, but there are still some issues. Please have the author review the attached materials.

Reviewer #2: Please perform a minor revision. Does L2 increase tomato yield? Please also write increase yield in the abstract. Also, clarify the chemical nitrogen fertilization application in L2.

Please check out citations problem in the whole manuscript ‘’Error! Reference source not found.,Error! Reference source’’

Correct caption of Fig 1.Tomato growth stages.Including growth images of four periods�transplanting、seeding、florescence、harvest

Yield(kg/Plant) in the table, please could you add the yield in kg/ha ?

Note(s): “*” indicates a significant correlation at the p < 0.05 level, and “**” indicates a highly significant correlationat the p < 0.01 level. In Table, write full forms of Index. For example, with note, write: Note(s): “*” indicates a significant correlation at the p < 0.05 level, and “**” indicates a highly significant correlationat the p < 0.01 level. U1 is xyz, U2 is xyz, ………..F6 is xyz.

Table 10. Scores and rankings of Tier 1 indicators. Write note For T1, T2, T3,

In conclusion, please clearly state which treatment combination is the optimum choice for the highest yield and quality of Tomato.

**********

what does this mean?). If published, this will include your full peer review and any attached files.

If you choose “no”, your identity will remain anonymous but your review may still be made public.

**Do you want your identity to be public for this peer review?**  For information about this choice, including consent withdrawal, please see our Privacy Policy

Reviewer #1: No

Reviewer #2: No

**********

[NOTE: If reviewer comments were submitted as an attachment file, they will be attached to this email and accessible via the submission site. Please log into your account, locate the manuscript record, and check for the action link "View Attachments". If this link does not appear, there are no attachment files.]

To ensure your figures meet our technical requirements, please review our figure guidelines: https://journals.plos.org/plosone/s/figures

You may also use PLOS’s free figure tool, NAAS, to help you prepare publication quality figures: https://journals.plos.org/plosone/s/figures#loc-tools-for-figure-preparation.

NAAS will assess whether your figures meet our technical requirements by comparing each figure against our figure specifications.

Attachment Submitted filename: PONE-D-25-44070_R1-Reviewer comments.docx

# Author comment: Author response to Decision Letter 2

**DOI:** 10.1371/journal.pone.0354927.r004

7 May 2026

Response to Reviewer 1comments

Point.1: Some references in the manuscript display “[Error! Reference source not found.,]”, the author needs to verify and revise.

Response 1: Thank you very much for your suggestion. The citation format of references has been revised.

Point.2: The abstract states three-way ANOVA, but the methods section states one-way ANOVA. The statistical model used needs to be consistent and clearly defined.

Response 2: Thank you very much for your suggestion. The statistical models in the paper have been kept consistent.

Point.3: The experimental design lacks reproducibility. Key information such as irrigation triggering method, single and cumulative irrigation volume, number of topdressing applications, and specific timing is still insufficiently detailed.

Response 3: Thank you very much for your suggestion. The key experimental information has been supplemented and explained in the sections "Test Materials and Experimental Design" and "Environmental Conditions and Water Consumption in Solar Greenhouse".

Point.4: Experimental replication is unclear. The text does not clearly state how many independent replicates each treatment has, or whether plots are statistical units.

Response 4: Thank you very much for your suggestion. We apologize for the unclear description of experimental replication. Each treatment included three independent biological replicates, and each plot was regarded as the basic statistical unit for ANOVA and multiple comparison analysis. Relevant details have been clearly supplemented in the Materials and Methods section of the revised manuscript.

Point.5: The description of the comprehensive evaluation method is too simplistic. PCA and entropy-weighted TOPSIS are key bases for deriving the “optimal treatment” but the related calculation procedures are not clearly explained.

Response 5: Thank you very much for your suggestion. We sincerely appreciate the constructive suggestion. To clarify the comprehensive evaluation procedures, we have supplemented the detailed calculation formulas and implementation steps of Principal Component Analysis (PCA) and Entropy-weighted TOPSIS in the Data Analysis section of the revised manuscript. The complete calculation process including data standardization, eigenvalue solving, weight determination, distance calculation and comprehensive scoring has been clearly presented, which fully supports the reliability of the optimal treatment screening result.

Point.6: Figures and tables still need optimization. The text “Fig 7. Comprehensive ranking of different treatments.” is not very readable; it is recommended to present the ranking and scores in a more intuitive way.

Response 6: Thank you very much for your suggestion. The figure is replaced by a more intuitive table.

Point.7: It is recommended that a native English speaker polish the entire text to ensure accurate terminology and fluent sentences.

Response 7: Thank you very much for your suggestion. The entire manuscript has been carefully polished and revised by a professional native English editing service to ensure accurate scientific terminology, proper grammar, and fluent expression. We believe the language quality of the revised manuscript has been significantly improved.

Response to Reviewer 2comments

Point.1: Please perform a minor revision. Does L2 increase tomato yield? Please also write increase yield in the abstract. Also, clarify the chemical nitrogen fertilization application in L2.

Response 1: Thank you very much for your suggestion.L2 can increase tomato yield, and relevant explanations of yield increase have been supplemented in the abstract. The application of chemical nitrogen fertilizer under each L2 treatment can be found in Table 2 in the text.

Point.2: Please check out citations problem in the whole manuscript ‘’Error! Reference source not found.,Error! Reference source’’

Response 2: Thank you very much for your suggestion. The citation format of references has been revised.

Point.3: Correct caption of Fig 1.Tomato growth stages.Including growth images of four periods�transplanting、seeding、florescence、harvest

Response 3: Thank you very much for your suggestion. Both the images and the caption in Figure 1 have been revised.

Point.4: Yield(kg/Plant) in the table, please could you add the yield in kg/ha ?

Response 4:We appreciate your valuable suggestion. However, we do not plan to modify the table to add the yield in kg/ha, as the current unit (kg/Plant) is more consistent with the research focus and data analysis logic of this study.

Point.5: Note(s): “*” indicates a significant correlation at the p < 0.05 level, and “**” indicates a highly significant correlationat the p < 0.01 level. In Table, write full forms of Index. For example, with note, write: Note(s): “*” indicates a significant correlation at the p < 0.05 level, and “**” indicates a highly significant correlationat the p < 0.01 level. U1 is xyz, U2 is xyz, ………..F6 is xyz.

Response 5: Thank you very much for your suggestion. The notes for Table 7 have been revised and completed.

Point.6: Table 10. Scores and rankings of Tier 1 indicators. Write note For T1, T2, T3,In conclusion, please clearly state which treatment combination is the optimum choice for the highest yield and quality of Tomato.

Response 6: Thank you very much for your suggestion. The notes for T1, T2, and T3 in Table 10 have been added. Information on the optimal treatment combination has been supplemented in the conclusion.

Attachment Submitted filename: new Response to Reviewers.docx

# Peer review: Decision Letter 2

**DOI:** 10.1371/journal.pone.0354927.r005

**Authors:** Umesh Sharma

1 Jun 2026

Dear Dr. li,

Thank you for submitting your manuscript to PLOS ONE. After careful consideration, we feel that it has merit but does not fully meet PLOS ONE’s publication criteria as it currently stands. Therefore, we invite you to submit a revised version of the manuscript that addresses the points raised during the review process.

Please submit your revised manuscript by Jul 16 2026 11:59PM. If you will need more time than this to complete your revisions, please reply to this message or contact the journal office at plosone@plos.org. When you're ready to submit your revision, log on to https://www.editorialmanager.com/pone/ and select the 'Submissions Needing Revision' folder to locate your manuscript file.

- A letter that responds to each point raised by the academic editor and reviewer(s). You should upload this letter as a separate file labeled 'Response to Reviewers'.
- A marked-up copy of your manuscript that highlights changes made to the original version. You should upload this as a separate file labeled 'Revised Manuscript with Track Changes'.
- An unmarked version of your revised paper without tracked changes. You should upload this as a separate file labeled 'Manuscript'.

If you would like to make changes to your financial disclosure, please include your updated statement in your cover letter. Guidelines for resubmitting your figure files are available below the reviewer comments at the end of this letter.

If applicable, we recommend that you deposit your laboratory protocols in protocols.io to enhance the reproducibility of your results. Protocols.io assigns your protocol its own identifier (DOI) so that it can be cited independently in the future. For instructions see: https://journals.plos.org/plosone/s/submission-guidelines#loc-laboratory-protocols. Additionally, PLOS ONE offers an option for publishing peer-reviewed Lab Protocol articles, which describe protocols hosted on protocols.io. Read more information on sharing protocols at https://plos.org/protocols?utm_medium=editorial-email&utm_source=authorletters&utm_campaign=protocols.

As the corresponding author, your ORCID iD is verified in the submission system and will appear in the published article. PLOS supports the use of ORCID, and we encourage all coauthors to register for an ORCID iD and use it as well. Please encourage your coauthors to verify their ORCID iD within the submission system before final acceptance, as unverified ORCID iDs will not appear in the published article. *Only*  the individual author can complete the verification step; PLOS staff *cannot*  verify ORCID iDs on behalf of authors.

We look forward to receiving your revised manuscript.

Kind regards,

Umesh Sharma

Academic Editor

PLOS One

Journal Requirements:

1. If the reviewer comments include a recommendation to cite specific previously published works, please review and evaluate these publications to determine whether they are relevant and should be cited. There is no requirement to cite these works unless the editor has indicated otherwise.

2. Please review your reference list to ensure that it is complete and correct. If you have cited papers that have been retracted, please include the rationale for doing so in the manuscript text, or remove these references and replace them with relevant current references. Any changes to the reference list should be mentioned in the rebuttal letter that accompanies your revised manuscript. If you need to cite a retracted article, indicate the article’s retracted status in the References list and also include a citation and full reference for the retraction notice.

Reviewers' comments:

Reviewer's Responses to Questions

**Comments to the Author**

Reviewer #1: (No Response)

Reviewer #2: All comments have been addressed

**********

2. Is the manuscript technically sound, and do the data support the conclusions?

Reviewer #1: Yes

Reviewer #2: Yes

**********

3. Has the statistical analysis been performed appropriately and rigorously?

Reviewer #1: Yes

Reviewer #2: Yes

**********

4. Have the authors made all data underlying the findings in their manuscript fully available?

The PLOS Data policy

Reviewer #1: Yes

Reviewer #2: Yes

**********

5. Is the manuscript presented in an intelligible fashion and written in standard English?

Reviewer #1: Yes

Reviewer #2: Yes

**********

Reviewer #1: The manuscript has been revised and improved based on the experts' opinions, and has adequately answered the experts' questions. However, there are still some doubts. Please review the reviewer's comments.

Reviewer #2: The revision is satisfactory. I have no additional comments. Authors should check the final manuscript carefully to avoid any errors during revisions.

**********

what does this mean?). If published, this will include your full peer review and any attached files.

If you choose “no”, your identity will remain anonymous but your review may still be made public.

**Do you want your identity to be public for this peer review?**  For information about this choice, including consent withdrawal, please see our Privacy Policy

Reviewer #1: No

Reviewer #2: No

**********

[NOTE: If reviewer comments were submitted as an attachment file, they will be attached to this email and accessible via the submission site. Please log into your account, locate the manuscript record, and check for the action link "View Attachments". If this link does not appear, there are no attachment files.]

To ensure your figures meet our technical requirements, please review our figure guidelines: https://journals.plos.org/plosone/s/figures

You may also use PLOS’s free figure tool, NAAS, to help you prepare publication quality figures: https://journals.plos.org/plosone/s/figures#loc-tools-for-figure-preparation.

NAAS will assess whether your figures meet our technical requirements by comparing each figure against our figure specifications.

Attachment Submitted filename: Reviewer Comments.docx

# Author comment: Author response to Decision Letter 3

**DOI:** 10.1371/journal.pone.0354927.r006

8 Jun 2026

Point.1: The authors measured soil field capacity, but the specific values are not provided in the text. Please supplement this information.

Response 1: Thank you very much for your suggestion.Field capacity data have been added to the manuscript.

Point.2: In Table 1, the soil type is given as loam, but the specific particle size distribution is not provided. Please provide this information.

Response 2: Thank you very much for your suggestion. The soil particle size data have been supplemented.

Point.3: The basis for the organic fertilizer application rates is missing. Please provide the rationale for the chosen rates.

Response 3: Thank you very much for your suggestion.Fertilization was performed in accordance with the Chinese agricultural industry standard Guidance for the Fertilization Control Techniques of Protected Vegetables (NY/T 3832-2021). The grading criteria for soil fertility specified in this standard are as follows:The table is provided in the attached reply document.

According to the soil test data in Table 1, the soil organic matter content corresponds to low fertility. Fertilizer application rates of N, P and K were determined referring to the above standard following the same calculation principle. Related explanations have been supplemented in the manuscript.

Point.4: In Equation (1), "total" should be written as a subscript.

Response 4: Thank you very much for your suggestion.The word "total" in Equation (1) has been changed to subscript form.

Point.5: Although transpiration rate and net photosynthetic rate showed no significant differences among treatments, intercellular CO₂ concentration and stomatal conductance did show significant differences. Please examine whether this has been explained in the Discussion section.

Response 5: Thank you very much for your suggestion.We have added corresponding explanatory content in the Discussion section.

Point.6: Lines 374–377 state: "However, except for a few treatments, there were no obvious differences in photosynthetic indices between different treatments, which may be attributed to the imprecise timing of photosynthetic measurements, leading to convergent leaf photosynthetic capacity under multi-factor coupling." Attributing the lack of differences to measurement timing seems somewhat tenuous. Is there any literature to support this statement?

Response 6: Thank you very much for your suggestion.Relevant explanations have been supplemented in the manuscript, and literatures supporting these conclusions have been cited in the reference list.

Point.7: In the Conclusions, the phrase "setting the soil irrigation level at 85% FC to 90% FC" is not accurate. Should it instead read as setting the upper limit of irrigation at 85–90% FC?

Response 7: Thank you very much for your suggestion.This expression has been revised to “setting the upper limit of irrigation at 85–90% FC” in the Conclusions as suggested.

Attachment Submitted filename: Response to Reviewer.pdf

# Peer review: Decision Letter 3

**DOI:** 10.1371/journal.pone.0354927.r007

**Authors:** Umesh Sharma

15 Jul 2026

Effects of different irrigation lower limits and combined application of reduced nitrogen with organic fertilizer on spring-season tomatoes (Solanum lycopersicum )

PONE-D-25-44070R3

Dear Dr. Haijie Li,

We’re pleased to inform you that your manuscript has been judged scientifically suitable for publication and will be formally accepted for publication once it meets all outstanding technical requirements.

Within one week, you’ll receive an e-mail detailing the required amendments. When these have been addressed, you’ll receive a formal acceptance letter and your manuscript will be scheduled for publication.

An invoice will be generated when your article is formally accepted. Please note, if your institution has a publishing partnership with PLOS and your article meets the relevant criteria, all or part of your publication costs will be covered. Please make sure your user information is up-to-date by logging into Editorial Manager at Editorial Manager® and clicking the ‘Update My Information' link at the top of the page. For questions related to billing, please contact billing support.

If your institution or institutions have a press office, please notify them about your upcoming paper to help maximize its impact. If they’ll be preparing press materials, please inform our press team as soon as possible -- no later than 48 hours after receiving the formal acceptance. Your manuscript will remain under strict press embargo until 2 pm Eastern Time on the date of publication. For more information, please contact onepress@plos.org.

Kind regards,

Umesh Sharma

Academic Editor

PLOS One

Additional Editor Comments (optional):

Reviewers' comments:

Reviewer's Responses to Questions

**Comments to the Author**

Reviewer #1: All comments have been addressed

**********

2. Is the manuscript technically sound, and do the data support the conclusions?

Reviewer #1: Yes

**********

3. Has the statistical analysis been performed appropriately and rigorously?

Reviewer #1: Yes

**********

4. Have the authors made all data underlying the findings in their manuscript fully available?

The PLOS Data policy

Reviewer #1: Yes

**********

5. Is the manuscript presented in an intelligible fashion and written in standard English?

Reviewer #1: Yes

**********

Reviewer #1: The author has made satisfactory revisions to the manuscript and addressed all of my questions. The data analysis method is reasonable and the results are correct. The language of the manuscript meets the requirements.

**********

what does this mean?). If published, this will include your full peer review and any attached files.

If you choose “no”, your identity will remain anonymous but your review may still be made public.

**Do you want your identity to be public for this peer review?**  For information about this choice, including consent withdrawal, please see our Privacy Policy

Reviewer #1: No

**********

# Editor report: Acceptance letter

**DOI:** 10.1371/journal.pone.0354927.r008

**Authors:** Umesh Sharma

PONE-D-25-44070R3

PLOS One

Dear Dr. li,

I'm pleased to inform you that your manuscript has been deemed suitable for publication in PLOS One. Congratulations! Your manuscript is now being handed over to our production team.

At this stage, our production department will prepare your paper for publication. This includes ensuring the following:

* All references, tables, and figures are properly cited

* All relevant supporting information is included in the manuscript submission,

* There are no issues that prevent the paper from being properly typeset

You will receive further instructions from the production team, including instructions on how to review your proof when it is ready. Please keep in mind that we are working through a large volume of accepted articles, so please give us a few days to review your paper and let you know the next and final steps.

Lastly, if your institution or institutions have a press office, please let them know about your upcoming paper now to help maximize its impact. If they'll be preparing press materials, please inform our press team within the next 48 hours. Your manuscript will remain under strict press embargo until 2 pm Eastern Time on the date of publication. For more information, please contact onepress@plos.org.

You will receive an invoice from PLOS for your publication fee after your manuscript has reached the completed accept phase. If you receive an email requesting payment before acceptance or for any other service, this may be a phishing scheme. Learn how to identify phishing emails and protect your accounts at https://explore.plos.org/phishing.

If we can help with anything else, please email us at customercare@plos.org.

Thank you for submitting your work to PLOS ONE and supporting open access.

Kind regards,

PLOS ONE Editorial Office Staff

on behalf of

Dr. Umesh Sharma

Academic Editor

PLOS One
